# Striatal dopamine signals reflect perceived cue–action–outcome associations in mice

**DOI:** 10.1038/s41593-023-01567-2

**Published:** 2024-01-30

**Authors:** Tobias W. Bernklau, Beatrice Righetti, Leonie S. Mehrke, Simon N. Jacob

**Affiliations:** 1 Translational Neurotechnology Laboratory, Department of Neurosurgery, Klinikum rechts der Isar, Technical University of Munich, Munich, Germany; 2 Graduate School of Systemic Neurosciences, Ludwig-Maximilians-University Munich, Munich, Germany

**Keywords:** Operant learning, Neural circuits

## Abstract

Striatal dopamine drives associative learning by acting as a teaching signal. Much work has focused on simple learning paradigms, including Pavlovian and instrumental learning. However, higher cognition requires that animals generate internal concepts of their environment, where sensory stimuli, actions and outcomes become flexibly associated. Here, we performed fiber photometry dopamine measurements across the striatum of male mice as they learned cue–action–outcome associations based on implicit and changing task rules. Reinforcement learning models of the behavioral and dopamine data showed that rule changes lead to adjustments of learned cue–action–outcome associations. After rule changes, mice discarded learned associations and reset outcome expectations. Cue- and outcome-triggered dopamine signals became uncoupled and dependent on the adopted behavioral strategy. As mice learned the new association, coupling between cue- and outcome-triggered dopamine signals and task performance re-emerged. Our results suggest that dopaminergic reward prediction errors reflect an agent’s perceived locus of control.

## Abstract (web summary)

Using dopamine photometry and reinforcement learning models in mice flexibly acquiring cue–action–outcome associations with rule switches, Bernklau et al. show that striatal dopamine reflects an animal’s current understanding of their task.

## Main

Forming associations between sensory stimuli in the environment and the outcomes of one’s own actions is a foundation of intelligent behavior and higher cognition. As one of the major neuromodulators in the brain, dopamine drives associative learning by acting as a teaching signal. Phasic activity of midbrain dopamine neurons indicates the difference between predicted and actual outcomes and corresponds to the reward prediction error (RPE) in reinforcement learning models, which is used to update state and action values^1,2^. This dopaminergic teaching signal modulates synaptic plasticity of cortical inputs to the striatum, an essential structure for reinforcement learning and a major projection target of dopamine neurons^3–5^. The dopamine RPE hypothesis is supported by a large body of evidence comprising recordings of dopamine neuron activity and dopamine release^6–9^ as well as causal optogenetic manipulations^10–13^. Much work has focused on the dopaminergic mechanisms of simple learning paradigms, such as Pavlovian or bandit tasks, in which outcome predictions are determined by experimentally manipulated reward magnitudes and probabilities. However, higher cognition requires an agent to actively generate concepts of its environment, in which sensory cues, actions and outcomes become associated. This entails that outcome predictions are conditional on one’s own actions and do not passively track the statistics of externally controlled outcomes. Yet, despite the well-established role of dopamine in other higher cognitive functions^14,15^, little is known about the role of dopamine in the acquisition of these more complex associations.

Fundamental insights into dopaminergic mechanisms of learning have typically been revealed in two groups of tasks. First, in Pavlovian or stimulus–outcome learning, phasic dopamine activity shifts from responding to the reward early in learning to responding to the reward-predicting cue after learning^16–20^, as predicted by reinforcement learning models^1,21^. In this setting, cue-triggered predictive dopamine signals and outcome-triggered reinforcing dopamine signals are determined by externally set reward expectations and are therefore strongly coupled to each other in the form of an inverse relationship. Second, in instrumental learning, outcomes are action dependent, and predictions are conditional on deliberate choices and internal representations thereof. In these tasks, learning is mostly defined not as the initial acquisition of associations but as adaptations to dynamic changes in probabilistic outcome contingencies during well-trained behavior. For example, in the commonly used bandit task, a classical reinforcement learning task, animals learn to select the most valuable action from two or more available options^22–25^. A successful behavioral strategy in bandit tasks is to repeat previous actions until changes in outcome contingencies are detected. Bandit choices thus do not require cue-based decisions but depend on the integration of trial outcome history^25^. Even tasks that include cue-based decisions use probabilistic outcome manipulations to drive learning^26–29^. As Pavlovian tasks, probabilistic paradigms produce coupling of dopamine signals through externally set variables that impose strict limits on the likelihood of obtaining desired outcomes, with little room for modification by one’s own actions.

Here, we addressed the role of dopamine in the acquisition of non-probabilistic three-term cue–action–outcome associations, where only the animals’ internal representations of the contingencies varied across learning of a task rule. We trained head-fixed male mice in an auditory decision-making task with implicit rule switches to elicit the repeated formation of new associations. We performed direct photometric measurements of dopamine in the ventral striatum (VS), dorsomedial striatum (DMS) and dorsolateral striatum (DLS) during all stages of task acquisition using fiber photometry and constructed reinforcement learning models of the behavioral and dopaminergic data. We found that rule changes prompted the animals to cut learned associations and reset outcome expectations, resulting in dissociations of cue- and outcome-triggered dopamine signals that depended on the adopted behavioral response strategy. As the animals rediscovered the impact of their own actions, reward predictions for the different trial events were recoupled, indicating an increased understanding of the current task. Our results expand the current understanding of the role of dopamine in learning, showing that the extent to which an agent is knowingly involved in determining task outcomes is reflected in distinct dopaminergic signatures.

## Results

### Mice learn cue–action–outcome associations

**Fig. 1:**
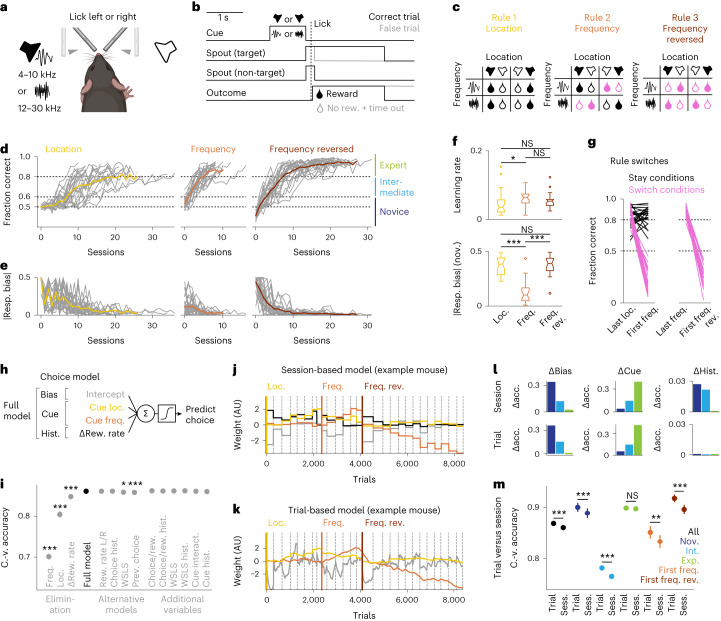
Behavioral task and choice modeling. **a**, Schematic of the behavioral setup (created with Biorender.com and used with permission). **b**, Task structure; rew., reward. **c**, Conditions and rule switches. **d**, Fraction of correct trials per session for *n* = 26 animals (gray) and the average across animals (color). **e**, Absolute response bias (resp. bias). **f**, Top, learning rate (Extended Data Fig. 1b; location (Loc.) versus frequency (Freq.), *P* = 0.026; location versus frequency reversed (Freq. rev.), *P* = 0.342; frequency versus frequency reversed, *P* = 0.592). Bottom, absolute response bias in novice (nov.) sessions and photometry sessions only (location versus frequency, *P* = 1.0 × 10^−5^; location versus frequency reversed, *P* = 1.0; frequency versus frequency reversed, *P* = 1.0 × 10^−5^). Data were analyzed by two-sided Wilcoxon signed-rank tests with Bonferroni correction. Box plot centers represent the median, box limits represent the upper/lower quartiles, whiskers represent 1.5× the interquartile range, and points represent outliers. **g**, Fraction of correct trials for the first 100 trials of the last session of the previous task rule and the following rule switch session split by stay and switch conditions. **h**, Logistic regression model. **i**, Cross-validated (C.-v.) prediction accuracy of the full model versus reduced/alternative models, photometry sessions only. Data were analyzed by two-sided Wilcoxon signed-rank tests (*n* = 26 animals) with a Holm–Bonferroni correction (cue frequency, *P* = 1.1 × 10^−4^; cue location, *P* = 1.1 × 10^−4^; ∆reward rate (∆Rew. rate), *P* = 1.3 × 10^−4^; reward rate left/right (L/R) instead, *P* = 1.0; choice history (Choice hist.) instead, *P* = 1.0; win–stay–lose–switch (WSLS) instead, *P* = 0.017; previous choice (Prev. choice) instead, *P* = 2.6 × 10^−4^; choice/reward (choice/rew.), *P* = 0.245; choice/reward history (choice/rew. hist.), *P* = 1.0; win–stay–lose–switch, *P* = 0.654; win–stay–lose–switch history (WSLS hist.), *P* = 0.184; cue interaction (cue interact.), *P* = 1.0; cue history (cue hist.), *P* = 0.209). **j**, Weight trajectories of session-based models across trials (example animal). The dashed gray lines represent session boundaries; AU, arbitrary units. **k**, Same as **j** for trial-based models. **l**, Difference in cross-validated prediction accuracy (Δacc.) by regressor group (Extended Data Fig. 2). **m**, Cross-validated prediction accuracy of the trial-based cue-only model versus the session-based full model; sess., session. Data were analyzed by two-sided Wilcoxon signed-rank tests (*n* = 26 animals) with a Holm–Bonferroni correction (all, 5.0 × 10^−5^; novice, *P* = 5.0 × 10^−5^; intermediate (Int.), *P* = 1.4 × 10^−4^; expert (Exp.), *P* = 0.062; first frequency (First freq.), *P* = 1.8 × 10^−3^; first frequency reversed (First freq. rev.), *P* = 5.2 × 10^−5^). Error bars represent s.e.m. across animals; NS, not significant. Source data

To study the acquisition of three-term cue–action–outcome associations, we developed an auditory decision-making task with rule switches for head-fixed mice (Fig. 1a–c). Mice learned implicit, uncued task rules in a non-probabilistic (that is, deterministic) environment by trial and error. After presentation of an auditory noise stimulus as an instruction cue, two drinking spouts were moved into reach of the tongue, and mice had to lick the left or right spout to retrieve a water reward (Fig. 1b). Rewards were only dispensed after a correct lick. Whether a trial was correct or false depended on the current task rule, which first required animals to follow cue location (left or right), then cue frequency (low or high) and finally cue frequency with reversed cue–action mapping (Fig. 1c).

**Extended Data Fig. 1:**
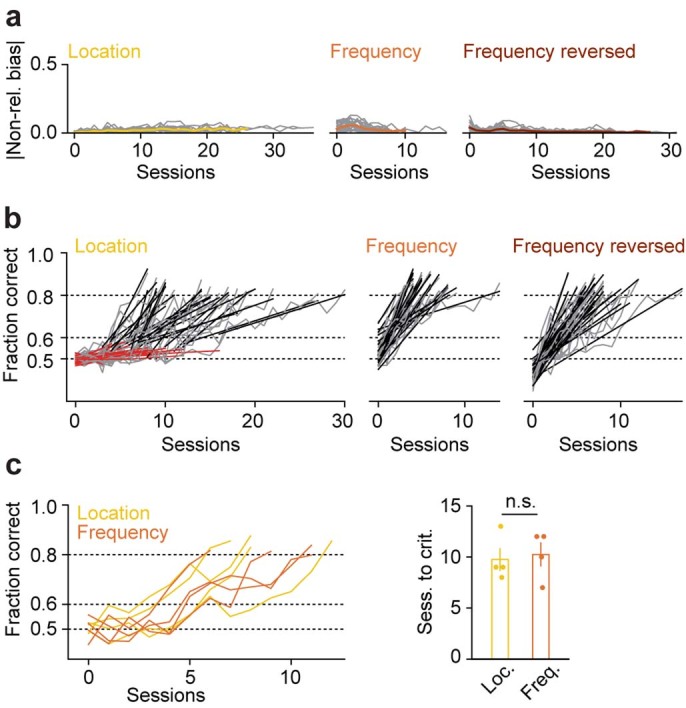
Pilot control study and details of behavioral analysis. **a**, Average absolute response bias per session for *n* = 26 animals (gray) and average across animals (color) for currently non-relevant cue dimension. **b**, Average fraction of correct trials per session up to the first criterion session for *n* = 26 animals for each task rule (gray) and linear fit to average performance across sessions from first session in task to first criterion (expert) session. Two fits for location task rule (red/black, see Methods). **c**, Left, average fraction of correct trials per session and per animal (*n* = 4 animals for location rule and *n* = 4 animals for frequency rule). Right, average number of sessions to criterion per task rule. Mean ± s.e.m. across animals. Two-sided permutation test, *P* = 0.886. Source data

Mice gradually learned the task rules across several sessions and then relearned when rules were switched after criterion performance had been reached (Fig. 1d). In the first sessions of the first task rule, mice chose a strategy of licking only at one spout (response bias) before they explored the other spout and gradually switched strategy to follow the instruction cue (Fig. 1e). Mice did not have biases with respect to the currently non-relevant cue dimension (Extended Data Fig. 1a). A linear fit of the learning rate revealed a small, yet significant, difference between the location rule and the frequency rule (Fig. 1f, top, and Extended Data Fig. 1b), suggesting that animals learned to learn. Additional nonspecific learning and habituation processes that had an impact on the learning rate in the first task rule cannot be excluded. In pilot experiments, we found no difference in the number of sessions to criterion between the location and frequency rule when both were used as first rules in naive animals, indicating that there was no difference between the two cue dimensions per se (Extended Data Fig. 1c). Analysis of behavioral performance after rule switches showed that animals perseverated and initially followed the previous task rule, resulting in unchanged high accuracy in ‘stay’ conditions (that is, conditions for which the correct response was the same under the new rule; 50% of trials after the first rule switch) and a substantial drop in accuracy in ‘switch’ conditions (that is, conditions for which the correct response was different under the new rule; 50% of trials after the first rule switch and 100% of trials after the second rule switch; Fig. 1g). Given the lower success rate, mice reverted to a simple response bias strategy after the second rule switch but not after the first rule switch (Fig. 1f, bottom).

### Mice use learning stage-dependent decision-making strategies

**Extended Data Fig. 2:**
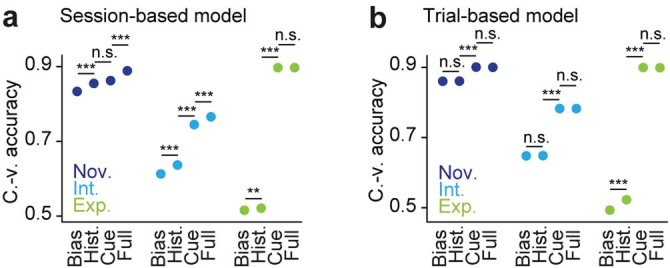
Choice model performance. **a**, Cross-validated prediction accuracy of the full session-based model compared to reduced models, averaged across sessions, split by performance levels. Two-sided Wilcoxon signed-rank tests (*n* = 26 animals) with Holm-Bonferroni correction for multiple comparisons. Novice: Bias vs. History, *P* = 2.8 × 10^−4^; History vs. Cue, *P* = 0.970; Cue vs. Full, *P* = 1.4 × 10^−4^. Intermediate: Bias vs. History, *P* = 1.4 × 10^−4^; History vs. Cue, *P* = 7.5 × 10^−5^; Cue vs. Full, *P* = 1.4 × 10^−4^. Expert: Bias vs. History, *P* = 0.008; History vs. Cue, *P* = 7.5 × 10^−5^; Cue vs. Full, *P* = 1.0. Markers and error bars show mean and standard error of the mean across sessions, respectively. **b**, Same as **a**, but for trial-based model. Novice: Bias vs. History, *P* = 1.0; History vs. Cue, *P* = 7.5 × 10^−5^; Cue vs. Full, *P* = 1.0. Intermediate: Bias vs. History, *P* = 1.0; History vs. Cue, *P* = 7.5 × 10^−5^; Cue vs. Full, *P* = 1.0. Expert: Bias vs. History, *P* = 7.5 × 10^−5^; History vs. Cue, *P* = 7.5 × 10^−5^; Cue vs. Full, *P* = 0.721. *P < 0.05, **P < 0.01, ***P < 0.001. Source data

To further characterize the animals’ strategies and detect possible additional contributions of trial history, we used logistic regression models to predict mouse choices in each trial^30–34^. We first fitted models per session using regressors for the response bias, the instruction cue and various regressors representing trial history-related effects (Fig. 1h). The final model was selected by cross-validation such that removing regressors degraded model accuracy, whereas adding or replacing trial history-related regressors did not improve model accuracy (Fig. 1i). The weight trajectories of the session-based model showed changes in strategy across sessions, with weights for the cue regressors progressively diverging from 0 across learning and weights for bias and history regressors converging to 0 across learning (Fig. 1j). To capture intrasession dynamics, we next used a trial-based model (Fig. 1k) with fluctuating weights across trials^30^. To assess the importance of regressors across training stages, which we defined as novice (below 60% correct trials per session), intermediate (between 60% and 80% correct trials) and expert (above 80% correct trials overall and above 60% correct trials in each condition; Fig. 1d), we examined the difference in prediction accuracy between models with and without particular regressors (Fig. 1l and Extended Data Fig. 2). In the session-based model, the bias and trial history regressors lost importance across learning and were no longer relevant in expert sessions, whereas the cue regressor gained importance and was the only relevant regressor in expert sessions (Fig. 1l, top). In the trial-based model, bias and cue regressors behaved similar to that in the session-based model, whereas the trial history regressor was not relevant, regardless of the training stage (Fig. 1l, bottom). Even without a trial history-related regressor, the trial-based model performed better than the full session-based model, especially in volatile sessions after rule switches but not in expert sessions when trial history was irrelevant (Fig. 1m). Together, these results suggest that history effects were sufficiently explained by fluctuations in response bias, which in the trial-based model were captured by fluctuating weights of the intercept across trials.

In summary, across learning, mice transitioned from a response bias strategy to an instruction cue strategy, showing that they gradually learned that outcomes are contingent on context-dependent actions and are not externally determined. As experts, mice solely relied on the instruction cue to guide their choices and were unaffected by response side biases or trial history.

### Cue and outcome dopamine signals evolve differently

**Fig. 2:**
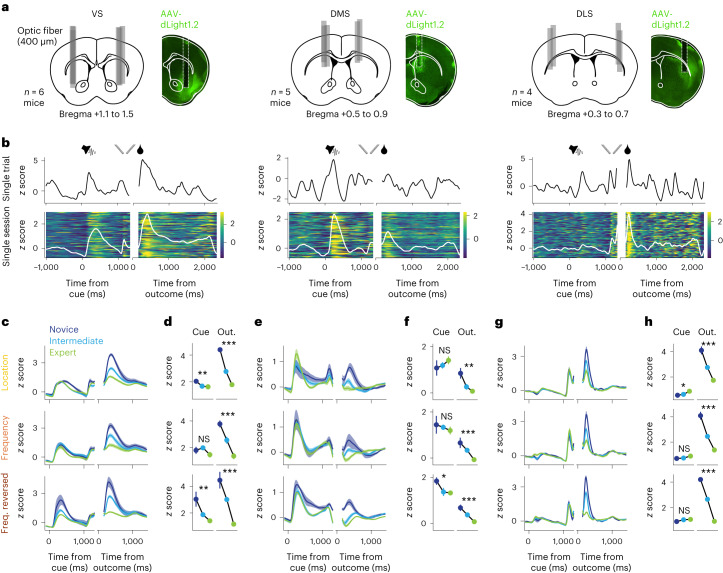
Dopamine signals across the striatum during task acquisition. **a**, Approximate implant locations of optic fibers for all animals (left) and one example animal (right) for three subregions of the striatum (VS, *n* = 6 animals; DMS, *n* = 5 animals; DLS, *n* = 4 animals). Left, schematics of coronal sections of the mouse brain with approximate fiber placements (gray bars). Right, histological example images of dLight fluorescence, overlayed with a mouse brain atlas schematic (solid white lines) and approximate fiber placement (dashed white lines). **b**, Top, normalized fluorescence of example trials for all subregions aligned to cue and outcome. Bottom, normalized fluorescence in example intermediate sessions (first 100 correct trials). False-color plots show the fluorescence in each trial (the first trial is on the bottom). White lines represent average fluorescence. **c**, Average normalized dLight fluorescence in the VS in correct trials split by performance levels for all task rules. For the location task, only late novice sessions were included to account for distinct learning stages (see Extended Data Fig. 1b and also Fig. 3a–c for early novice sessions). Data are shown as mean ± s.e.m. across sessions. **d**, Average normalized dLight fluorescence peaks in the VS across trial epochs (cue and outcome (out.)) in correct trials split by performance levels; data are shown as mean ± s.e.m. across sessions and were analyzed by Kruskal–Wallis tests across pooled sessions; *P < 0.05; **P < 0.01; ***P < 0.001. See Supplementary Table 1 for exact *P* values. Results were confirmed by linear mixed model analyses with animal as the grouping factor (see Supplementary Table 2). **e**,**f**, Same as **c** (**e**) and **d** (**f**) for the DMS. **g**,**h**, Same as **c** (**g**) and **d** (**h**) for the DLS. Source data

**Extended Data Fig. 3:**
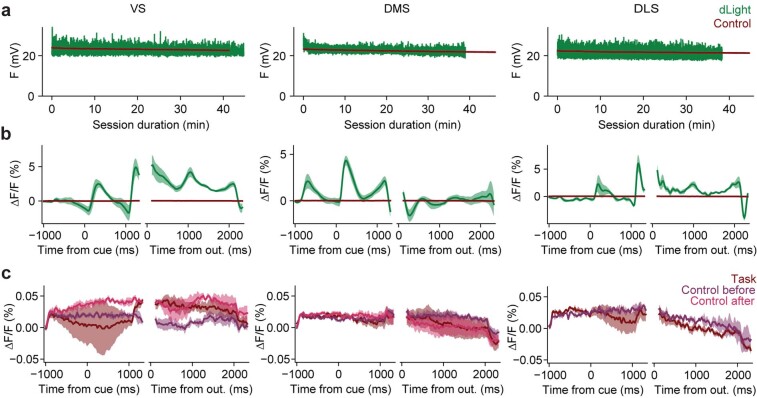
Photometry control measurements. **a**, Raw fluorescence in an example session with dLight measurement (green) or control measurement (red). **b**, Relative fluorescence for the last task sessions with dLight photometry (green) and a task session after the experiment with autofluorescence control (red). Mean ± s.e.m. across animals (VS, *n* = 6 animals, DMS, *n* = 5 animals, DLS *n* = 4 animals). **c**, Same as **b**, but only control signals from three different sessions before and after the experiment (‘Task’: last task session, ‘Control before’: pre-training session before task learning, ‘Control after’: pre-training session after task learning). Source data

To investigate how striatal dopamine signals triggered by the cue and outcome evolve during learning, we performed direct fiber photometric dopamine measurements with the fluorescent sensor dLight1.2 (ref. ^35^) expressed in the VS (*n* = 6), DMS (*n* = 5) and DLS (*n* = 4), each of which have been shown to have distinct functional roles in associative learning^36^ (Fig. 2a). Potential differences in dopaminergic signaling across projection targets are best examined by measuring dopamine concentrations directly in the target region because they can diverge from somatic activity^23^. Control measurements indicated that movement artifacts were negligible in our head-fixed preparation (Extended Data Fig. 3).

**Extended Data Fig. 4:**
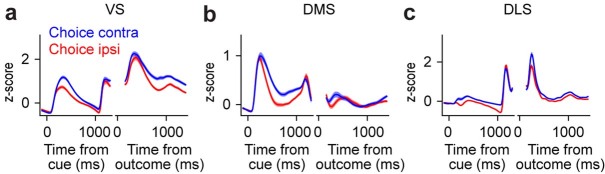
Lateralization of dopamine signals. **a-c**, Average normalized dLight fluorescence in VS (**a**), DMS (**b**), and DLS (**c**) in correct trials split by choice ipsilateral and contralateral to implanted hemisphere. Source data

Trials elicited robust phasic dopamine responses with differences across subregions regarding temporal dynamics and responsiveness to particular events (Fig. 2b). In the VS, all events triggered large dopamine transients. In the DMS, responses to the instruction cue dominated responses to the outcome, whereas the opposite pattern was observed in the DLS (Fig. 2c–h). In line with previous reports^25,29^, we found a mild effect of lateralization, with stronger cue-triggered and outcome-triggered dopamine signals for choices contralateral to the implanted hemisphere than for choices ipsilateral to the implanted hemisphere (Extended Data Fig. 4).

We compared trial-averaged dopamine transients between the novice, intermediate and expert training stages (Fig. 2c–h). Dopamine signals triggered by obtained rewards decreased with performance levels across all subregions and task rules (Fig. 2d,f,h), in line with dopamine RPEs^1^. By stark contrast, dopamine signals triggered by the instruction cue did not increase accordingly as seen in Pavlovian^19,37,38^ or probabilistic tasks^28,29^. The absence of a hypothesized inverse relationship between cue and outcome dopamine indicated that these signals were not consistently coupled across learning.

### Predictive dopamine signals depend on behavioral strategy

The lack of increase of cue-triggered dopamine signals across learning was most evident in the location and frequency reversed tasks, during which animals applied a simple response bias strategy and did not align their actions with the instruction cue but instead licked predominantly either the left or the right spout (Fig. 1f, bottom). We therefore investigated dopamine signals in early training stages to explore the extent to which dopamine signatures reflected how the animals internally conceptualized the current task.

**Fig. 3:**
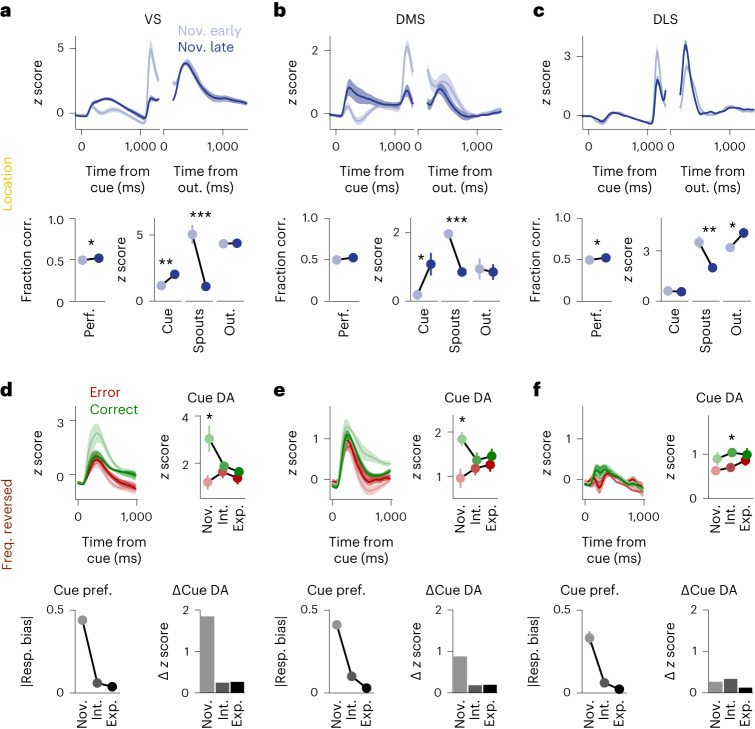
Predictive dopamine signals across behavioral strategies. **a**, Top, average normalized dLight fluorescence in the VS in correct trials split by early and late novice sessions during the location rule. Data are shown as mean ± s.e.m. across sessions. Bottom left, average fraction of correct trials (fraction corr.) for early and late novice sessions. Bottom right, average normalized dLight fluorescence peaks in trial epochs cue, spouts and outcome (out.) in correct trials split by early and late novice sessions (*n* = 12/12 sessions for early/late). Data were analyzed by Kruskal–Wallis tests across pooled sessions (performance (perf.), *P* = 0.021; cue, *P* = 0.002; spouts, *P* = 1.4 × 10^−4^; outcome, *P* = 0.525). **b**, Same as **a** for the DMS (*n* = 10/10 sessions; performance, *P* = 0.059; cue, *P* = 0.028; spouts, *P* = 1.6 × 10^−4^; outcome, *P* = 1.0). **c**, Same as **a** for the DLS (*n* = 12/12 sessions; performance, *P* = 0.018; cue, *P* = 0.817; spouts, *P* = 0.003; outcome, *P* = 0.033). **d**, dLight fluorescence and response bias across performance levels in the frequency reversed task. Top left, average normalized dLight fluorescence in the cue epoch in the VS split by error and correct trials. Top right, average normalized dLight fluorescence peaks in the cue epoch. Data were analyzed by two-sided Wilcoxon rank-sum tests across pooled sessions with a Bonferroni correction for multiple comparisons (novice, *n* = 6 sessions, *P* = 0.046; intermediate, *n* = 9 sessions, *P* = 0.566; expert, *n* = 8 sessions, *P* = 0.234). Bottom left, average absolute response bias. Bottom right, average normalized dLight fluorescence peaks in the cue epoch in the VS; the difference between error and correct trials across performance levels is shown; DA, dopamine; pref., preference. **e**, Same as **d** for the DMS (novice, *n* = 10 sessions, *P* = 0.021; intermediate, *n* = 11 sessions, *P* = 0.590; expert, *n* = 10 sessions, *P* = 1.0). **f**, Same as **d** for the DLS (novice, *n* = 11 sessions, *P* = 0.196; intermediate, *n* = 14 sessions, *P* = 0.034; expert, *n* = 8 sessions, *P* = 1.0). See Supplementary Table 2 for linear mixed model analyses with animal as the grouping factor. Source data

**Extended Data Fig. 5:**
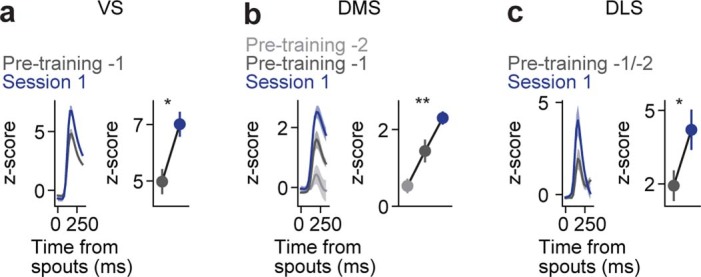
Spout-triggered dopamine signals during pre-training. **a**, Left, average normalized dLight fluorescence in VS in correct trials in spouts epoch during pre-training sessions and first task sessions. Mean ± s.e.m across sessions. Right, average normalized dLight fluorescence peak heights in spouts epoch. Kruskal-Wallis test across *n* = 6 animals. *P* = 0.016. **b**, same as **a**, but for DMS. *P* = 0.008, *n* = 5 animals. **c**, same as **a**, but for DLS. *P* = 0.043, *n* = 4 animals. *P < 0.05, **P < 0.01. Source data

VS dopamine signals triggered by the reward dispensing spouts built up during pretraining, in which rewards were retrieved from the spouts in the absence of instruction cues (Extended Data Fig. 5). This suggests that the presentation of the spouts initially acted as a Pavlovian cue. As the animals subsequently entered the location task and the sole presentation of the spouts was no longer predictive of reward, these spout-triggered dopamine signals quickly decreased (Fig. 3a). In parallel, dopamine signals triggered by the cue in correct trials increased, indicating that the dopamine signal advanced in time to the instruction cue (Fig. 3a). Thus, in these trials in which the instructed side coincided with an animal’s preference for licking that same spout, the instruction cue served as a Pavlovian cue. At the same time, there was no change in outcome-triggered dopamine, and task performance was still close to chance level (Fig. 3a), corroborating the notion that predictive and reinforcing signals were uncoupled when a cue–action–outcome association had not yet been formed. This effect was comparable in the DMS (Fig. 3b) but was less prominent in the DLS (Fig. 3c).

Similarly, in novice sessions in the frequency reversed task, mice again adopted a strong response bias strategy (Fig. 1f, bottom) and received rewards mainly from their preferred spout. We compared correct and error trials to reveal potential differences in cue-triggered dopamine signaling, which would indicate passive tracking of what the animals perceived to be externally controlled reward rates. Cue-triggered dopamine signals in the VS were larger in correct trials than in error trials. This difference decreased across learning in conjunction with a decrease in response bias (Fig. 3d). Thus, the cue-triggered dopamine signature initially resembled a passive Pavlovian-like prediction of rewarded versus unrewarded trials as in the early stages of the location task, which vanished once the animals abandoned the response bias strategy (see also Fig. 2d). The decrease of cue-triggered dopamine across learning was again comparable in the DMS (Fig. 3e, see also Fig. 2f) but was absent in the DLS (Fig. 3f), where cue dopamine signals were small relative to outcome dopamine signals (Fig. 2h).

### Reward predictions are recoupled during learning

Next, we leveraged the fact that the first rule switch produced strong performance differences between stay and switch trials due to perseverative behavior (Fig. 1g). This allowed us to investigate how differences in internal outcome expectations (high and low for stay and switch trials, respectively) affected dopaminergic reward predictions triggered by the different trial events across learning.

**Fig. 4:**
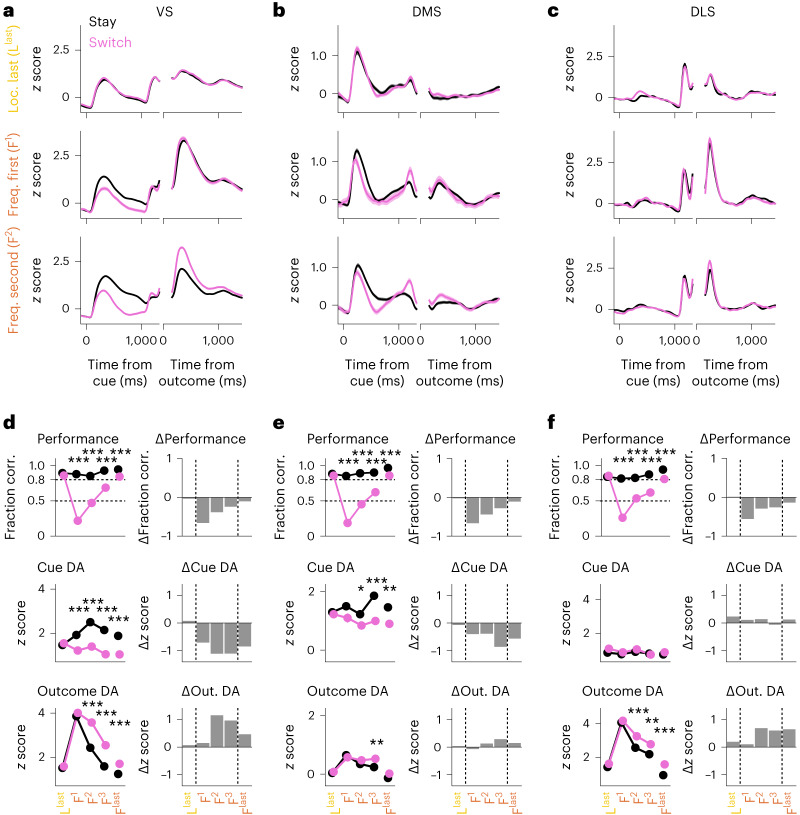
Uncoupling and recoupling of cue and outcome dopamine signals after rule switch. **a**–**c**, Average normalized dLight fluorescence across the VS (**a**), DMS (**b**) and DLS (**c**) in correct trials split by stay and switch conditions for the last location session (L^last^) and first two frequency sessions (F^1^ and F^2^). Data are shown as mean ± s.e.m. across trials. **d**, Top left, fraction of correct trials for the last location session (L^last^), the first three frequency sessions (F^1^, F^2^ and F^3^) and the last frequency session (F^last^) split by stay and switch conditions. Top right, difference between fraction correct in stay and switch conditions. Middle and bottom, average normalized dLight fluorescence peaks in trial epochs cue (middle) and outcome (bottom) in correct trials split by stay and switch conditions (left) and the difference between stay and switch conditions (right). Data are shown as mean ± s.e.m. across trials and were analyzed by two-sided Wilcoxon rank-sum tests across pooled trials and a Bonferroni correction for multiple comparisons; *P < 0.05; **P < 0.01; ***P < 0.001. See Supplementary Table 1 for exact *P* values and Supplementary Table 2 for linear mixed model analyses with animal as the grouping factor. **e**, Same as **d** for the DMS. **f**, Same as **d** for the DLS. Source data

Immediately after the first rule switch, the VS cue-triggered dopamine signal in stay trials increased relative to that in switch trials (Fig. 4a,d, middle), reflecting the difference in behavioral performance. Surprisingly, however, the outcome-triggered dopamine signal was the same in stay and switch conditions (Fig. 4a,d, middle and bottom, respectively) despite the strong behavioral differences. Thus, the outcome-triggered dopamine signal increased not only in switch trials, in which rewards were now unexpected, but also in stay trials, in which mice were able to successfully apply the previous rule and rewards should have been well expected. Only in the second session after the rule switch did outcome-triggered dopamine signals start to again reflect the difference in behavioral performance, that is, outcome dopamine signals were smaller in stay trials and larger in switch trials (Fig. 4a,d, bottom). As learning progressed and behavioral performance increased, the differences in dopamine signals between stay and switch trials decreased (Fig. 4d). These findings suggest that after the rule switch, mice had reset their outcome expectations. Thus, in novice sessions, the cue–action–outcome association was corrupted, and mice had not yet incorporated their actions into the reward prediction. As animals acquired the new association, coupling of outcome dopamine signals to task performance and cue dopamine was reinstated.

Overall, compared to the VS, these dopamine signatures were qualitatively similar in the DMS and DLS. In the DMS, there was a focus on the cue dopamine signal with minor differences in the outcome dopamine signal (Fig. 4b,e), whereas in the DLS, there were no differences in the cue dopamine signal with a focus on the outcome dopamine signal (Fig. 4c,f).

### Negative prediction errors are not scaled with performance

Dopamine ‘dips’ (negative RPEs) during epochs in which rewards unexpectedly do not occur are a hallmark of dopaminergic teaching signals^1,39^ and are thought to be similarly important for guiding future behavior as positive RPEs^40^. We therefore now focused on outcomes in error trials to (1) investigate whether negative deflections in dopamine were present in our task and (2) whether potential decreases in dopamine were equally affected by task performance as increases in dopamine (Fig. 2c–h). We considered two hypotheses. First, outcome-triggered dopamine signals in error trials could be negatively shifted parallel to correct trials, that is, increase in amplitude across learning. Such a parallel shift, as seen in probabilistic tasks^37,39^, would assume similar internal states for correct and error trials and indicate passive tracking of external reward rates. Second, outcome-triggered dopamine signals in error trials could mirror those in correct trials, that is, decrease in amplitude across learning. This would be in line with the concept of belief states^41^ and comparable to confidence-dependent dopamine signals observed in perceptual tasks^29^.

**Fig. 5:**
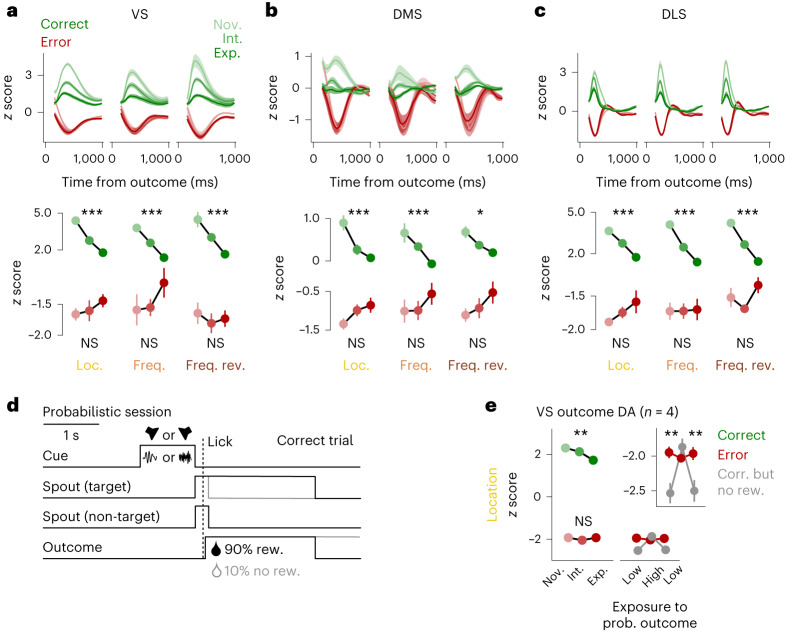
Dopamine signals following positive and negative outcomes. **a**, Top, average normalized dLight fluorescence in the outcome epoch in the VS during the location (left), frequency (middle) and frequency reversed rule (right) split by correct and error trials across performance levels. Data are shown as mean ± s.e.m. across sessions. Bottom, average normalized dLight fluorescence peaks or valleys in the outcome epoch. Data were analyzed by Kruskal–Wallis tests across pooled sessions (location (*n* = 25/15/12 sessions for novice/intermediate/expert): correct *P* = 5.6 × 10^−9^ and error *P* = 0.829; frequency (*n* = 6/9/11 session): correct *P* = 2.2 × 10^−4^ and error *P* = 0.536; frequency reversed (*n* = 6/9/8 sessions): correct *P* = 4.7 × 10^−4^ and error *P* = 0.838). **b**, Same as **a** for the DMS (location (*n* = 21/22/10 sessions): correct *P* = 7.8 × 10^−4^ and error *P* = 0.158; frequency (*n* = 5/12/10 sessions): correct *P* = 1.4 × 10^−4^ and error *P* = 0.512; frequency reversed (*n* = 10/11/10 sessions): correct *P* = 0.022 and error *P* = 0.293). **c**, Same as **a** for the DLS (location (*n* = 26/24/8 sessions): correct *P* = 3.0 × 10^−6^ and error *P* = 0.215; frequency (*n* = 5/11/7 sessions): correct *P* = 4.2 × 10^−4^ and error *P* = 0.965; frequency reversed (*n* = 11/14/8 sessions): correct *P* = 4.3 × 10^−6^ and error *P* = 0.091). **d**, Schematic of correct trials in the probabilistic session (10% without rewards). **e**, Left, same as **a**, bottom left, for *n* = 4 new animals. Data were analyzed by Kruskal–Wallis tests across pooled sessions (*n* = 29/32/18 sessions; correct *P* = 0.007 and error *P* = 0.575). Right, probabilistic sessions following at least seven deterministic expert sessions or only one deterministic session (low or high recent exposure to probabilistic outcome, respectively). Data shown are for correct but non-rewarded trials within the first 50 trials of the session. Data shown in the inset were analyzed by two-sided Wilcoxon rank-sum tests across pooled trials with Bonferroni corrections for multiple comparisons (low, *P* = 0.005; high, *P* = 0.223; low, *P* = 0.004). Data are shown as mean ± s.e.m. across trials. See Supplementary Table 2 for linear mixed model analyses with animal as the grouping factor. Source data

We found consistent negative dopamine signals in error trials without rewards during all task rules and training stages (Fig. 5a–c). Negative outcome-triggered dopamine signals in error trials were not inversely scaled with task performance. In line with our second hypothesis, the amplitude of dopamine dips decreased across learning, following a mirrored rather than parallel course of the positive dopamine peaks. The same signatures were present in all subregions. However, the scaling of dopamine dips across learning did not reach significance. This might be a consequence of a smaller dynamic range of negative deflections in fluorescent dopamine measurements, leading to an attenuation of the amplitude of dopamine dips. To rule out that this experimental limitation did not allow us to detect a parallel shift of negative compared to positive signals, we designed a new task condition to suppress dopaminergic activity below the levels of the original tasks. In a separate group of animals implanted in the VS (*n* = 4) and trained on the location task rule, rewards in expert sessions were omitted in 10% of correct trials and administered in 10% of error trials (probabilistic sessions; Fig. 5d). Outcome-triggered dopamine signals in this group of animals were comparable to the original VS data (Fig. 5e, left, compare to Fig. 5a). Importantly, the absence of reward despite correct responses triggered larger negative dopamine deflections than the absence of reward following incorrect responses, that is, in regular unprovoked error trials (Fig. 5e, right, left data points). This effect disappeared with repeated exposure to probabilistic outcomes (Fig. 5e, right, middle data points) and re-emerged again after several sessions without this outcome manipulation (that is, after several deterministic sessions; Fig. 5e, right, right data points).

In summary, these findings showed that despite the reduced dynamic range at the signal floor, differences in dopamine signal reductions could still be measured. Our results suggest that negative dopamine RPEs decreased across learning, arguing that behavioral errors had less impact on learning in expert animals. Furthermore, the two task variants together demonstrated that negative dopamine RPEs depended on whether reward omission was endogenously produced by or externally forced upon the animal (deterministic and probabilistic sessions, respectively). These results add to the notion that dopamine signals do not simply passively track reward rates when they are under the control of the animals’ actions, in agreement with evolving belief states regarding the nature of the task.

### Temporal difference learning captures dopamine signatures

Our results so far indicate that dopamine signals reflect the extent to which an animal has incorporated its own actions into a mechanistic understanding of its environment. To gain more detailed insights into the neural computations underlying the link between an agent’s actions and dopamine RPEs, we constructed temporal difference reinforcement learning (TDRL) models. We chose generative models of state-action value learning to simulate the key behavioral and dopamine signatures observed in the data. In these frameworks, an agent transitions through a sequence of states and learns to take appropriate actions with the goal of maximizing future reward. We tested on-policy (SARSA, state-action-reward-state-action) and off-policy Q-learning algorithms, which differ regarding the ability to learn from chosen and not chosen actions^42–44^.

**Fig. 6:**
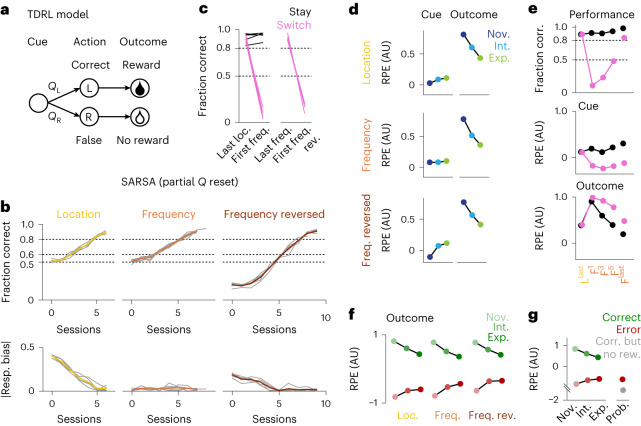
TDRL model of behavioral and dopamine signatures. **a**, State space exemplified for one condition in one environment. See Extended Data Fig. 6a for the full state space; *Q*_L_/*Q*_R_, state-action values for the left/right action in the cue state, respectively. **b**, Simulation of SARSA agents with partial *Q* reset (that is, only *Q*_L_/*Q*_R_ values in cue states were retained after rule switches). Top, average fraction of correct trials per session for *n* = 5 agents (gray) and average across agents (color) for each task rule. Bottom, average absolute response bias per session. **c**, Average fraction of correct trials per agent for the first 100 trials of the last session of the previous task rule and the following rule switch session split by stay and switch conditions. **d**, Average RPE in the cue epoch (left) and outcome epoch (right) in correct trials split by performance levels for all task rules. **e**, Top, fraction of correct trials for the last location session (L^last^), first frequency session (F^1^), selected intermediate frequency sessions and last frequency session (F^last^) split by stay and switch conditions. Middle and bottom, average RPE in the cue (middle) and outcome (bottom) trial epochs in correct trials split by stay and switch conditions. **f**, Average RPE in the outcome epoch split by correct and error trials across performance levels for all task rules. **g**, Left, average RPE in the outcome epoch split by correct and error trials across performance levels during acquisition of the location task. Right, average RPE in the outcome epoch during the probabilistic session. Source data

**Extended Data Fig. 6:**
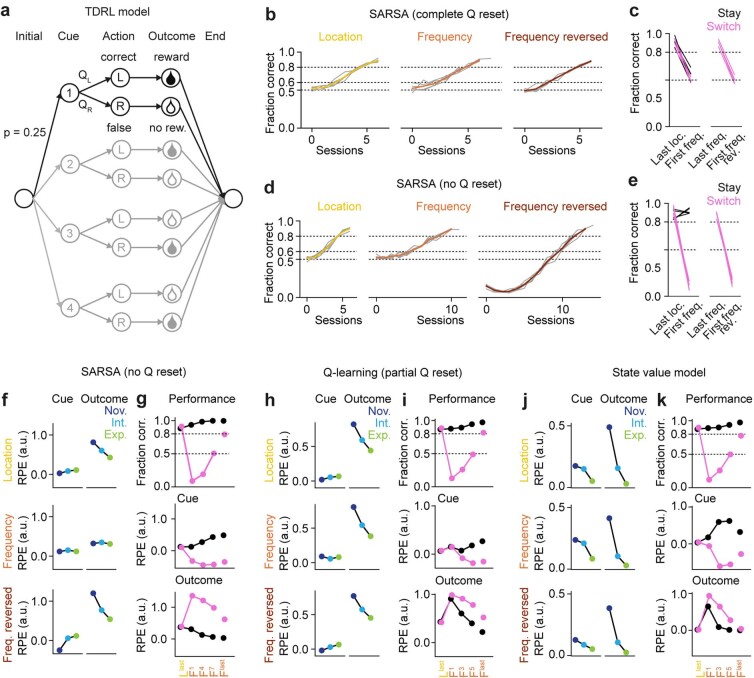
Full TDRL model state space and different implementations of learning. **a**, Full state space of TDRL models, exemplified for one environment (that is, task rule) and highlighting one condition (black). State transitions were determined by the environment except in cue states, where the agent transitioned to one of two action states depending on its choice. For deterministic state transitions only one state-action value is shown. Q_L/R_, state-action values for the left/right action in the cue state. **b**, Simulation of SARSA agents with complete Q reset (that is, no Q-values were retained after rule switches). Top, average fraction of correct trials per session for *n* = 5 agents (gray) and average across agents (color) for each task rule. **c**, Average fraction of correct trials per agent for the first 100 trials of the last session of the previous task rule and the following rule switch session, split by stay and switch conditions. **d**, **e**, same as **b**, **c**, but for SARSA agents without Q reset (that is, all Q-values were retained after rule switches). **f**, Average reward prediction error (RPE) for SARSA agents without Q reset in the cue epoch (left) and outcome epoch (right) in correct trials, split by performance levels for all task rules. **g**, Top, fraction of correct trials for last location session (L^last^), first frequency session (F^1^), selected intermediate frequency sessions, and the last frequency session (F^last^), split by stay and switch conditions. Middle and bottom, average RPE in trial epochs cue (middle) and outcome (bottom) in correct trials, split by stay and switch conditions. **h**, **i**, same as **f**, **g**, but for Q-learning model with partial Q reset (that is, only Q_L/R_ values in cue states were retained after rule switches). **j**, **k**, same as **f**, **g**, but showing state RPEs obtained from the SARSA partial Q reset model. Source data

We constructed the task environment such that an agent learns and updates *Q* values that are used to guide choices and predict future outcomes (Fig. 6a and Extended Data Fig. 6a). Experimental data showed that rule switches triggered an adjustment in the animals’ learned cue–action–outcome associations, as evidenced by a re-emergence of strong outcome dopamine signals across all conditions (Figs. 2 and 4). In our model, we therefore reset all *Q* values representing prediction of outcome but not *Q* values linking cues to actions (*Q*_L_ and *Q*_R_), which guide the choice. With this partial *Q* value reset, SARSA agents reproduced the animals’ behaviors well, capturing gradual learning of task rules and, in particular, perseveration following rule switches (Fig. 6b,c). Retaining choice-guiding information was crucial because resetting all *Q* values did not achieve this result (Extended Data Fig. 6b,c).

RPEs obtained from this model also reproduced the dopamine signatures across learning (Fig. 6d,e), especially with respect to outcomes across subregions and task rules. Specifically, RPEs triggered by the outcome increased after rule switches (Fig. 6d) equally for stay and switch trials (Fig. 6e, bottom). RPEs triggered by the cue were separated for stay and switch trials directly after the first rule switch (Fig. 6e, middle), recapitulating the uncoupling of cue and outcome dopamine signals observed in the data (Fig. 4). By contrast, in a model without *Q* value resets, in which the agents retained all learned outcome expectations, learning after rule switches was slower (Extended Data Fig. 6d,e), and RPEs did not match the dopamine data after the first rule switch. Specifically, outcome-triggered RPEs increased only in switch trials (Extended Data Fig. 6g) and remained equal when averaged across conditions (because there were more correct stay trials; Extended Data Fig. 6f, middle). Together, these results indicate that the rule switch and concomitant drop in performance caused animals to (partially) discard the learned associations, which was advantageous for learning.

We also tested an off-policy Q-learning model. This model failed to capture the characteristic uncoupling of cue-triggered and outcome-triggered RPEs that was present in the SARSA model with partial *Q* value reset (Extended Data Fig. 6h,i). This difference could be due to the deterministic nature of the task with infrequent changes in reward contingencies, which prompted animals to learn from chosen actions and did not encourage exploration.

Finally, modeled outcome RPEs, in particular the mirrored negative deflections with decreasing amplitudes across learning (Fig. 6f), resembled the relationship between dopamine dips and dopamine peaks in the experimental data (Fig. 5a–c). Simulating outcome RPEs in probabilistic sessions (Fig. 6g) also recapitulated the observed difference between endogenously produced and externally forced errors in experimentally obtained outcome dopamine signals (Fig. 5e).

Notably, modeled cue-triggered RPEs did not approximate VS and DMS cue dopamine signals in novice sessions in the location and frequency reversed task, in which the animals exhibited a strong response bias (Fig. 6d, top and bottom, compare to Fig. 2d,f). This was the case despite the fact that the response bias was explicitly modeled in the action selection process (see Methods), which indirectly affected RPEs. This finding corroborates the idea that the Pavlovian-like passive outcome predictions in early learning stages with simple response bias strategies (Fig. 3d,e) formed a separate mechanism that could not be accounted for by state-action value prediction errors. Fittingly, we found that pure state RPEs approximated the increased VS and DMS cue response in correct trials in novice sessions with strong response biases (Extended Data Fig. 6j). State RPEs did not match the dopamine data in the frequency task (Extended Data Fig. 6k), however, where the animals actively pursued the previous task rule without strong response biases and state-action RPEs explained the data well.

## Discussion

We studied dopamine signaling across the striatum as mice learned to integrate instruction cues, actions and outcomes in a task where reward expectations depended solely on the internal representation of the learned association but not on the statistics of externally controlled outcomes (that is, reward magnitudes and probabilities). Our findings suggest that dopamine RPEs reflect the animals’ understanding of the task.

Although outcome-triggered dopamine signals scaled with behavioral performance and decreased during learning, cue-triggered dopamine signals did not increase accordingly (Fig. 2). These results are in contrast to previous findings from Pavlovian or probabilistic tasks in which cue and outcome dopamine signals are inversely coupled^16–20,26–29^. The key difference in our study was that outcome probabilities were not externally set (with the exception of infrequent rule switches) but depended on task performance, which was under the control of the animals. However, our results do not contradict but complement previous results using other learning paradigms by providing evidence for a dopaminergic mechanism of associative learning in non-probabilistic environments. Our TDRL model captured the main behavioral and dopaminergic signatures of the present task (Fig. 6). In particular, modeling suggested an adjustment in the animals’ learned cue–action–outcome associations after rule switches. Indeed, the experimental data showed that outcome-triggered dopamine signals re-emerged after introduction of a new task rule and did so equally for stay and switch conditions after the first rule switch despite strong differences in behavioral performance, which should have triggered different RPEs. This disruption after rule switches could be modeled by a reset of *Q* values that was only partial and discarded outcome-predictive information but retained choice-guiding information. A reset of state-action values in the model resembles the discovery of new states, which might be segmented by large prediction errors, and has served as a useful explanation of behavioral responses to changing contexts^45,46^. Together, our experimental and modeling results thus indicate that the rule switches prompted animals to cut the learned cue–action–outcome association and rediscover the impact of their own actions, that is, the locus of control.

Emphasizing the central role of goal-directed actions in the behavior we studied, our results provide evidence that behavioral strategies shape dopaminergic signatures. Cue-triggered dopamine signals passively tracked external outcome statistics, that is, encoded Pavlovian-like reward prediction of rewarded versus unrewarded trials, only when cue–action–outcome associations were not yet established (Fig. 3). In these early learning stages, mice had not yet incorporated their own actions into concepts of the current task but exhibited a strong response side bias. Animals therefore experienced one side as more rewarding than the other and thus falsely attributed rewards to external factors. As a new association was actively formed, the locus of control shifted toward the animal as an active agent, which was accompanied by reduced passive tracking. TDRL modeling showed that active learning could be well approximated by state-action value learning (Fig. 6). Interestingly, passive tracking was not captured by state-action RPEs but could be modeled by pure state RPEs, which recapitulated the response bias-related dopamine cue responses. Together, these results suggest an additional mechanism that is triggered in situations with low perceived control over outcomes. Therefore, in light of recent findings regarding functional heterogeneity in dopamine neurons^47^, it is conceivable that signals associated with active and passive learning are mixed in striatal projections, for example, in the form of distributed RPE coding^48^.

After the first rule switch, cue-triggered dopamine was higher for stay conditions than for switch conditions in novice sessions (Fig. 4), which at first glance resembled passive tracking after the second rule switch (that is, difference in cue dopamine signals between correct and error trials in novice sessions). The key difference to novice sessions in the other tasks, however, was that the animals did not adopt a strong response bias strategy after the first rule switch but instead actively pursued the previous task rule. Fittingly, this dopamine signature was explained by state-action value prediction errors, specifically by those produced by an on-policy learning algorithm. Interestingly, on-policy SARSA and off-policy Q-learning predicted qualitatively different RPE signatures after the first rule switch. The dopamine data clearly favored SARSA (Fig. 6e versus Extended Data Fig. 6i). There is mixed evidence in the literature regarding what type of state-action value learning might be used by the brain^27,49^. SARSA and Q-learning differ most in exploratory trials (that is, trials in which the lower-valued action is selected by mistake). Correct switch trials after the first rule switch were exactly such trials, which is why the SARSA model, comparable to the data, showed separation between stay and switch trials for cue-triggered RPEs after the rule switch, representing the previously learned values. A correct response in a switch trial was a false response before the rule switch, so in a correct switch trial, a low-valued action was chosen by mistake. SARSA fitting better to the data could be rooted in the nature of the task. Q-learning is more optimal for exploration and, in contrast to SARSA, does not penalize choosing the lower-valued option for exploratory purposes. Our task did not incentivize exploration because rule switches and accompanying changes in contingencies were rare, unlike in probabilistic bandit tasks. In conjunction with the re-emergence of outcome RPEs uniformly in stay and switch conditions, signifying a reset of outcome expectancies after the rule switch, these SARSA-modeled cue RPEs reproduced the uncoupling observed in cue and outcome dopamine when the animals had to re-evaluate the consequences of their own actions.

In our non-probabilistic learning task, negative dopamine RPEs were not inversely scaled with task performance but tended to diminish across learning (Fig. 5). This is in contrast to parallel offsets between positive and negative dopamine RPEs, which reflect externally driven outcome expectations both during the delivery of rewards and the omission of rewards^37,39^ but is in line with belief states shaping dopamine RPEs^29,41^. We speculate that this result is rooted in the changing extent to which animals attribute outcomes to external versus internal factors, resulting in expectations that do not simply track external reward rate. When animals were experts and had a firm concept of the task, there was less need to update their behavioral strategy after errors, which may explain attenuated negative dopamine RPEs and is consistent with the lowered behavioral sensitivity to outcomes that is a characteristic of beginning habit formation after extensive training^36^. This notion was supported by our experimental finding that externally forced reward omissions produced stronger reductions in dopamine than own unforced errors (Fig. 5). This result also ruled out that a floor effect in the fluorescent measurements prevented the detection of a parallel shift of dopamine dips relative to dopamine peaks. The TDRL model reproduced both the upward ramping course of negative RPEs and the RPE differences for the two types of errors (Fig. 6), fitting to the on-policy SARSA agent already accounting for the (probably bad) choice in error trials when it processes the cue.

Despite strong similarities across striatal subregions in our study, we found differences between subregions regarding temporal dynamics^50^ and learning-related changes in dopamine signals. Although state-action value learning models captured the main dopamine signatures, individual aspects were differentially expressed in the different subregions. In the DLS, or sensorimotor striatum, we did not find large dopamine transients during the cue epoch but found them predominantly following outcomes. Dopamine signals in this subregion most closely resembled the action value prediction errors from the model, in agreement with a hypothesized involvement of the DLS in action learning and habit formation^36^. In contrast to a previous study that did not observe negative RPEs in this subregion^29^, we found similar dopamine dips in all striatal subregions, including the DLS. This might be explained by differences in the nature of the measured signals (axonal calcium activity versus dopamine release) or differences in the behavioral task. In the VS, or motivational striatum, cue-triggered dopamine signals most strongly reflected the Pavlovian-like encoding of rewarded and unrewarded trials in sessions with strong response bias and therefore most strongly deviated from modeled action value prediction errors, which is in line with the role of the VS in motivational functions and Pavlovian learning^36^. In the DMS, or associative striatum, cue-triggered dopamine signals were larger than reward-triggered dopamine signals, which were close to 0 in expert sessions. Absence of reward-triggered dopamine signals in the DMS has been reported previously in well-trained animals^25,29,51^. We found that reward-triggered dopamine signals are indeed present in the DMS in early training stages. Reward-related dopamine signals in the DMS have also been observed after changes in outcome contingencies^52^, matching the strong and transient modulation of outcome-triggered dopamine signals in the DMS after rule switches in our task. Taken together, DMS dopamine could be used to facilitate behavioral adaptations after rule switches, in agreement with the assumed role of the DMS in goal-directed behavior and behavioral flexibility^36,53^.

In conclusion, our findings provide insights into the dopaminergic signatures underlying the integration of sensory cues, actions and outcomes into a mechanistic understanding of the environment, which is the foundation of intelligent behavior. The principles of biological learning play a vital role in informing algorithms in artificial learning^54^. Future efforts should be directed at studying learning in increasingly naturalistic environments to foster fruitful interactions between the fields of biological and artificial intelligence^55^.

## Methods

### Animals

All animal procedures were authorized by the local government (Regierung von Oberbayern, license number ROB-55.2-2532.Vet_02-17-119). Animal health was examined and scored every day. Wild-type male mice (C57BL/6J, Charles River) were used for all experiments. Mice were 8–10 weeks old at the beginning of the experiments and were housed in single cages on a reverse 12-h light/12-h dark cycle (that is, dark during the day). Ambient humidity and temperature were set to 50% and 24 °C, respectively. Mice had ad libitum access to food and water except during behavioral experiments.

### Virus injection and fiber implantation

A total of 30 mice were implanted. Animals were initially anesthetized with 2% isoflurane and transferred to a stereotaxic frame (Neurostar), where isoflurane anesthesia was maintained at 0.8–1.5%. Analgesia (200 mg per kg (body weight) metamizol and 1.5 mg per kg (body weight) meloxicam) was injected subcutaneously. Body temperature was controlled with a thermometer and adjustable heating pad, and respiration was visually monitored. Hair above the skull was removed with a shaver, and the skin above the skull was disinfected with 70% ethanol. Local anesthetics (2% lidocaine solution) were injected subcutaneously, and the skin above the skull was excised. The skull was cleaned thoroughly with 0.9% sodium chloride solution and roughened with forceps in preparation for implantation. Guided by automated navigation software (Neurostar StereoDrive version 3.1.5) for correction of tilt and scaling of the skull, a 0.6-mm craniotomy was performed above the target location of virus injection and fiber implantation.

For expression of the fluorescent dopamine sensor dLight^35^, 200 nl of AAV5.hSyn.dLight1.2 (titer of 4 × 10^12^ genome copies per ml) was injected unilaterally with a glass capillary nanoinjector (Neurostar NanoW). pAAV-hSyn-dLight1.2 was a gift from L. Tian, UC Davis (Addgene viral prep 111068-AAV5; http://n2t.net/addgene:111068; RRID: Addgene_111068). The virus was injected in one of the following target regions: lateral VS (bregma +1.3 mm anterior, ±1.8 mm lateral, +4.3 mm ventral), DMS (bregma +0.7 mm anterior, ±1.3 mm lateral, +2.6 mm ventral) or DLS (bregma +0.5 mm anterior, ±2.5 mm lateral, +2.8 mm ventral). Seven mice were implanted in the VS, five mice were implanted in the DMS, and four mice were implanted in the DLS. One VS animal was excluded from dLight analysis due to low signal amplitude. Two VS animals were lost during training and completed only the first two tasks. Four additional mice were implanted in the VS for the probabilistic experiment (Fig. 5d,e). Left and right hemispheres were counterbalanced across animals for each target region.

For virus injection, a glass capillary with a tip diameter smaller than 50 µm was lowered and retracted at 0.5 mm min^–1^. The virus was injected at a rate of 50 nl min^–1^. The glass capillary was retracted after 10 min of diffusion time. Ready-to-implant 1.25-mm optic fiber ferrules (Thorlabs, CFMXD05 or CFMXD04) or equivalent custom-built ferrules (using Thorlabs FP400URT, CFX440 and NOA63) with a fiber diameter of 400 µm and numerical aperture of 0.5 were implanted 200 µm above the injection site. The length of the implanted fibers was approximately 0.5 to 1 mm longer than the dorsoventral implantation coordinate. To minimize tissue trauma, the fiber was iteratively lowered 200 µm and retracted 100 µm at a speed of 2 mm min^−1^ until the implantation site was reached. The ferrule was fixed to the skull with light-curing adhesive (OptiBond All-in-One) and dental cement (Tetric EvoFlow). A custom-made metal bar for head fixation was fixed to the skull posterior to the fiber implant. Animals received postoperative analgesia (1.5 mg per kg (body weight) meloxicam) for 3 d following the surgery.

### Histology

After behavioral and photometry experiments, animals were perfused with 4% paraformaldehyde. Brains were postfixed for 24 h with fiber implants in place. Brains were sliced in coronal sections at 120 µm using a vibratome and covered with mounting medium (VectaShield). Histological slices were imaged using a confocal microscope (Leica SP8) with tenfold magnification. Confocal images were overlayed with sections from the mouse brain atlas^56^ for verification of implantation locations.

### Behavior

#### Behavioral setup

Behavioral experiments were performed in sound-attenuated boxes (Med Associates). Mice were head fixed and rested in a body tube. Two drinking spouts were moved into and out of reach of the animals’ tongues using servo motors (Turnigy, TGY 313C) and a microcontroller (Arduino Uno Rev3). When positioned within reach of the tongue, the spouts were approximately 4–8 mm apart. Spout contacts of the tongue (that is, lick responses) were monitored as threshold crossings of the metal-to-water junction potential^57^. Water rewards were dispensed from the spouts using a TTL-controlled syringe pump (New Era Pump Systems, NE-500). Electrostatic speakers for ultrasonic sound production (Tucker-Davis Technologies, ED1 and ES1) were positioned 10 cm to the left and right of the animals’ ears at an angle of 15° toward the front. A monitor with a diagonal of 25.4 cm (Faytech, FT10TMB) was positioned 15 cm in front of the animals. The behavioral protocol was implemented using MATLAB (version R2018a) and the MATLAB toolbox MonkeyLogic (version 2, build 206)^58^ to control a data acquisition device (National Instruments, PCIe-6323) with a break-out panel (National Instruments, BNC-2090A). Time-stamped behavioral event codes were sent to the photometry recording system.

#### Controlled water protocol

After at least 3 d of postoperative recovery and 3 to 4 weeks of viral expression time, mice were administered a controlled water protocol to motivate them for behavioral experiments. Animals received water during daily training sessions in which they drank 1,000 to 1,500 µl of water. If mice drank less than 1,000 µl in a training session, they received additional water from a pipette. Animals were typically trained every day. Body weight and health scores were examined daily to ensure that body weight was maintained above 80% of the weight before the surgery.

#### Habituation

On the first day of habituation, mice were slowly accustomed to handling by the experimenter and were introduced to the body tube for head fixation. To reinforce the habituation, animals received water rewards from a pipette during handling. On the second or third day of habituation, mice were head-fixed in the setup and received free water rewards from one stationary licking spout.

#### Pretraining

For mice to learn to respond to two movable licking spouts, they received three to four sessions of pretraining in which only one of the two licking spouts was presented in a randomized order, and a water reward was released after an instrumental lick. After a maximum of four pretraining sessions, when mice consumed at least 800 µl of water during one session, they progressed to the full task.

#### Auditory decision-making task

All 30 implanted mice were trained to perform an instrumental response lick on one of two drinking spouts to obtain a water reward. Water rewards were only dispensed after a correct lick. The correct spout was indicated by an auditory instruction cue, which was either low-frequency band-pass-filtered white noise (4 to 8 kHz) or high-frequency band-pass-filtered white noise (16 to 32 kHz) at 75–80 dB sound pressure level. At the beginning of each trial, the monitor’s luminance was raised to indicate the trial start. The monitor was at background luminance between trials. After 1,000 ms, the auditory cue was played on one of the speakers for 1,000 ms. Following the offset of the cue, the two licking spouts were moved into reach of the animals’ tongues, and the response window started. In the response window, the first contact of the tongue with either of the two spouts was registered as the behavioral response. When the first lick was on the correct spout, a water reward of 5 µl was triggered to be dispensed from the target spout. The non-target spout was retracted immediately after a correct lick on the target spout. When the first lick in the response window was on the incorrect spout, both spouts were retracted, and an error time out of 4,000 ms was initiated in addition to the regular intertrial interval of 4,000 ms. In miss trials, when no lick was detected, both spouts were retracted, and the regular intertrial interval began after the 2,000-ms response window. Trials were presented in blocks of 32 trials in pseudorandomized order, that is, within a block, the conditions were drawn randomly without replacement.

#### Task rules

Animals were trained on three different implicit task rules based on the two instruction cue dimensions, location and frequency. Animals in the main experiment were first trained on the location rule and had to lick the left spout following a sound from the left speaker or the right spout following a sound from the right speaker to obtain a reward. Once animals had acquired the first rule, the rule was switched to the previously irrelevant frequency dimension, and animals had to lick the left spout following a low-frequency sound and the right spout following a high-frequency sound to obtain a reward. Finally, after animals had acquired the frequency rule, the rule was switched within the dimension of frequency, and animals had to lick the left spout following a high-frequency sound and the right spout following a low-frequency sound to obtain a reward. In probabilistic sessions (Fig. 5d), the contingencies were reversed in 10% of the trials such that a correct response triggered the outcome normally triggered in incorrect trials (that is, spout retraction and time out), whereas an incorrect response triggered the outcome normally triggered in correct trials (that is, reward).

#### Session durations

Behavioral sessions were terminated manually when miss trials due to satiety were noticed. Sessions were then truncated post hoc at the first miss trial after 90% of the trials and were further truncated if the performance dropped below 1.5 s.d. during the last 15% of trials before the first miss trial. Overall, this method resulted in 5.67% of trials being excluded. A negligible amount of miss trials (0.02% of all trials) were also excluded from the analysis. The average session duration after truncating was 330 trials (s.d., 77 trials).

To obtain quasicontinuous data points of dopamine measurements across the learning process, dopamine signals were recorded in every other behavioral session. Criterion performance for task acquisition was defined as at least 80% of correct trials per session and at least 60% of correct trials in each of the four conditions. Task rules were switched when animals had performed at least two photometry sessions at criterion performance, and the following behavior-only session was also a criterion session.

#### Response bias

Mice naturally developed idiosyncratic preferences for either of the two spouts. Two quantify this preference, a response bias index was calculated as (fraction correct left trials – fraction correct right trials)/(fraction correct left trials). The absolute response bias (|response bias|) was calculated as *|(*response bias – 0.5)| and thus ranged between 0 and 0.5, with larger values indicating larger absolute response biases independent of the response side. The response preference could be manipulated by adjusting the relative position of the two licking spouts, that is, positioning the non-preferred spout closer or the preferred spout further away. When extreme side preferences were observed, the spouts were positioned individually for each animal before the session to counteract their strong preference in previous sessions. In behavior-only sessions (that is, every other session), but not in photometry sessions, the response bias was monitored, and spout positions were adjusted online. The intrasession adjustments were not conducted during photometry sessions to keep the effort of reaching the spouts constant. Bias adjustments were only conducted in novice sessions during the first task rule but not in later sessions or after rule switches.

#### Learning rate

Learning rate was defined as the slope of a linear function fit to the performance curve across sessions from the first session in the task to the first criterion session. For the first task rule (location), two fits were used to account for the apparent slope increase in the learning curves after several novice sessions with strong response bias. The first fit was performed from the first session to the last novice session and the second fit from the first intermediate session to the first criterion (expert) session. Fitting two lines instead of one yielded a better fit for the location task rule.

#### Pilot experiments

In pilot experiments, the task rule for the initial task acquisition was varied to examine potential preferences for one or the other instruction cue dimension. The pilot experiment was identical to the main experiment, except for the following difference. In a fraction of trials, only the target spout was presented, forcing the animal to make a correct choice. These forced-choice trials were used in pilot experiments to speed up task acquisition, but not in the main experiment. In the pilot experiments, animals showed no difference in the initial task acquisition time between location and frequency rules, suggesting that animals did not prefer either instruction cue dimension per se.

### Fiber photometry

Fiber photometric signals were acquired with a two-channel analog optometer with amplification and filter modules (NPI Electronic, FOM-02 and LPBF-01GD). For dLight measurements, a 470-nm LED, 442- to 478-nm excitation and 500- to 530-nm emission filter and a 495-nm dichroic mirror were used. For control measurements, a 556-nm LED, 546- to 566-nm excitation filter, 589- to 625-nm emission filter and 573-nm dichroic mirror were used. The two channels were separated with a 532-nm dichroic mirror.

A low-autofluorescence patch cable (Thorlabs, FP400URT-CUSTOM) was connected to the implanted ceramic ferrule using a ceramic mating sleeve (Thorlabs, ADAL1). The excitation light intensity was set to 50 µW at the tip of the patch cable in all dLight photometry sessions. The transmission rate of the implanted ferrules was between 80% and 86%, as tested before implantation, resulting in an excitation intensity of 40 to 43 µW. The fluorescence signals were amplified and filtered in hardware with a gain of 100 and a low-pass filter at 100 Hz, digitized at a 1-kHz sampling rate using a data acquisition system (Plexon Omniplex version 1.18.3) and recorded together with behavioral time stamps.

Control photometry sessions were performed using the 556-nm channel. Because the excitation light in this control channel did not overlap with the excitation wavelength of dLight, the recorded signal was assumed to represent background autofluorescence independent of dLight activity. The light intensity was titrated individually for each animal to match the background fluorescence level in the control channel to the level of dLight recordings, resulting in intensities from 60 to 250 µW. Control recordings showed negligible amplitudes compared to dLight signals.

### Choice models

The session-based choice model was a logistic regression model. The probability of an animal’s choice ${\hat{y}}_{i}$ in each trial was modeled as a linear combination of predictors passed through a logistic function

$$\widehat{y}=\frac{1}{1+{e}^{-z}},$$

where

$$z=\sum _{p}{\beta }_{p}+{\beta }_{0},$$

and where ${\beta }_{p}$ is the regression weight for predictor *p*, and ${\beta }_{0}$ is an intercept, which represents a general tendency for a left or right response. Regressor weights were optimized by minimizing the negative log-likelihood function

$$J=-\frac{1}{m}\mathop{\sum }\limits_{i}^{m}y\,\log (\,{\widehat{y}}_{i})+(1-{y}_{i})\log (1-{\widehat{y}}_{i}),$$

where $m$ is the number of trials, ${y}_{i}$ is the actual choice in trial *i*, and ${\hat{y}}_{i}$ is the predicted probability of choice in trial $i$. Weights were optimized by gradient descent, and the optimization was stopped when the loss was below 1 × 10^−4^ or a maximum number of iterations was reached. Binary model predictions for choice were calculated by rounding the model probability $\hat{y}$ to 0 or 1. The model was validated using the Pyglmnet toolbox (version 1.1)^59^, which produced similar results.

The model was fit per session with tenfold cross-validation. The samples of all regressors were randomly split into training and test sets ten times, such that every sample appeared in the test set once. The predictions for all samples from the test sets were used to calculate the cross-validated prediction accuracy, which was used for model selection. Using likelihood-based measures, such as pseudo-*R*^2^, cross-validated bit per trial^34^ or information criteria (Bayesian information criterion and Akaike information criterion), for model selection produced similar results. Prediction accuracy was used for model evaluation due to intuitive interpretation. Elastic net regularization, including a grid search for the regularization parameters, did not qualitatively change the results. Therefore, no regularization was used.

Regressors for the final model were selected such that the prediction accuracy across all sessions was significantly reduced when a regressor was removed, and the prediction accuracy was not significantly enhanced when other regressors were added or alternative regressors were used. The following regressors were part of the final session-based choice model: cue location of the current trial (−1 for the left speaker and +1 for the right speaker), cue frequency of the current trial (−1 for the low-frequency sound and +1 for the high-frequency sound) and ∆reward rate (the difference of a ten-trial exponentially weighted average rate of rewards up to and including the previous trial calculated separately for each spout, ranging between −1 and 1). ∆Reward rate indicated whether a reward was expected more on the left spout or on the right spout according to the history of previous choices and rewards. Several alternative models were compared to the final model. The first alternative trial history model included the reward rates of the left and right spout (ten-trial exponentially weighted average of rewards up to and including the previous trial, ranging between 0 and 1). The second alternative trial history model included three predictors for the choice up to three trials back (−1 for the left choice and +1 for the right choice). The third alternative trial history model included two predictors representing a ‘win–stay’ and ‘lose–switch’ strategy, respectively. Win–stay was coded as −1 following a correct previous trial with a left choice, as +1 following a correct previous trial with a right choice and as 0 following a false previous trial. Lose–switch was coded as −1 following a false previous trial with a left choice, as +1 following a false previous trial with a right choice and as 0 following a correct previous trial. The fourth alternative trial history model included only the choice in the previous trial (−1 for left previous choice and +1 for right previous choice). The additional variables included the choice in the previous trial and previous trial outcome (−1 for no reward in the previous trial and +1 for reward in the previous trial) and the interaction thereof, the previous choice and outcome and interaction thereof up to three trials back, the win–stay and lose–switch predictors, the interaction of the cue predictors and the history of the cue up to three trials back. Input variables were all in the range between −1 and +1 and were not standardized to avoid mean centering and to ascertain interpretability of weight deviations from 0.

The trial-based choice model was fit using the PsyTrack toolbox^30^, which also models choice in a logistic regression. The PsyTrack model was fit per animal using optimized hyperparameters to allow for fluctuations of regressor weights throughout the learning process, both across trials and across sessions. The recommended default initial hyperparameters were used. Model predictions were again made with tenfold cross-validation to calculate the cross-validated prediction accuracy and compare the PsyTrack model to the custom session-based model. The same regressor variables as in the final session-based model were used.

### Data analysis

Fiber photometric signals were smoothed with a 50-ms running average and downsampled to 50 samples per s. For analysis of trial-related modulations of dLight, relative fluorescence ∆*F*/*F* was calculated by subtracting a baseline from every sample and dividing it by the baseline using the average amplitude of a 500-ms window before the trial start as a baseline. Using baselining methods that retain slow fluctuations across the session (for example, subtracting a polynomial fit or low-pass-filtered version of the whole-session signal) yielded qualitatively similar results. The ∆*F*/*F* signals were normalized using a robust *z* score (subtracting the median and dividing by the median absolute deviation) calculated for each session using the analyzed trial sections to account for differences in signal intensities across sessions and animals. Calculating the *z* score using only the baseline period or using the whole session did not change the main results.

### Statistics

No statistical methods were used to predetermine sample sizes, but our sample sizes are similar to those reported in previous publications^20,28^. For tests of statistical significance, sessions were pooled across animals when groups of sessions were compared (for example, comparisons across performance levels). Trials were pooled across sessions when different trial types were compared (for example, comparisons across stay and switch trials). The main results were not qualitatively different without pooling. Non-parametric tests were used for comparisons across two groups (Wilcoxon signed-rank/rank-sum test) and three or more groups (Kruskal–Wallis test), unless otherwise stated. Significance levels were adjusted for multiple comparisons (Bonferroni correction), as noted in the figure captions. To account for the hierarchical structure in the data, we performed additional linear mixed model analyses with fixed and random effects for all tests with pooled data using animal identity as the grouping factor for random intercepts and random slopes. Linear mixed models confirmed the main results (see Supplementary Table 2). Data are presented as mean ± s.e.m. across animals, sessions or trials, as specified in the figure captions. Box plot elements are defined in the following way: center lines represent the median values, box limits represent the upper and lower quartiles, whiskers represent 1.5× interquartile range, and points represent outliers. Significance levels are indicated as *P < 0.05, **P < 0.01 and ***P < 0.001 in all figures.

Dopamine peaks (positive or negative) were defined as the peak or valley with the larger prominence in a given time window. The time windows were selected to account for differences in dynamics of dopamine signals across trial epochs and striatal subregions. Peaks in the cue epoch were detected using windows of 1,000 ms after cue presentation for all subregions. Peaks in the outcome epoch were detected using a window of 840 ms for the VS, 740 ms for the DMS and 380 ms for the DLS. The windows for the outcome epoch started with a short latency after the instrumental lick (160 ms for the VS and DMS and 140 ms for the DLS) to account for latencies in reward delivery and kinetics of the fluorescent sensor. To avoid visual doubling of signals triggered by the presentation of the spouts, this latency was also used in all figures showing outcome-triggered dopamine signals. For the trial epoch of the spout movements, a window of 340 ms after the cue offset was used for all subregions. Differences in average dopamine levels were also calculated by simple averages across time windows instead of peak extraction, which produced qualitatively similar results. Further, main results did not depend on the exact window lengths.

Data were analyzed with Python (version 3.6.8) using the packages NumPy (version 1.16.4)^60^, SciPy (version 1.5.4)^61^, pandas (v0.25.1)^62^, matplotlib (v3.1.3)^63^, scikit-learn (version 0.22.1)^64^, statsmodels (version 0.11.1)^65^ and pymer4 (version 0.8.0)^66^ in addition to the toolboxes mentioned earlier.

Data collection and analysis were not performed blind to the conditions of the experiments. All animals underwent the same behavioral protocol. There was no random assignment to experimental groups.

### Reinforcement learning models

TDRL models of state-action values were used to simulate mouse behavior and RPEs in the form of TD errors. Because we were interested in mechanistic insights at a conceptual level, we did not perform formal quantitative fits to the data but instead designed the models’ environment and agent configurations to qualitatively capture the behavioral and neural signatures.

For each task rule, one task environment was constructed. The environments included five temporal states (initial, cue, action, outcome and end; Extended Data Fig. 6a). We used four cue states to represent the four conditions, eight action states to represent the combination of four conditions and two response sides (left and right) and eight outcome states to represent the combination of the four conditions and two outcomes (that is, correct or false). This resulted in a total of 22 states. In each episode representing a trial, the agent visited each of the temporal states. From the initial state, the agent transitioned to one of the four cue states with uniform probability. In the cue state, a left or right action could be selected. Missed actions were not modeled due to the negligibly small amount of miss trials in the data. Depending on the selected action, the agent transitioned to the left or right action state. The agent then transitioned to the reward state or the no-reward state depending on whether the action (selected during the cue state) was correct or false, respectively. Entering the reward state, a reward of 1 was obtained, whereas entering the no-reward state, a reward of −1 was obtained. For the modeling of probabilistic sessions (Fig. 6g), the environment was changed such that in 10% of trials, there was a transition from the correct action state to the no-reward state and a transition from the false action state to the reward state.

We tested two different standard state-action value learning algorithms: an on-policy variant of Q-learning (SARSA, state-action-reward-state-action^43^) and off-policy Q-learning^42^. The goal of the agent is to maximize the cumulative future reward

$${R}_{t}=\mathop{\sum}\limits_{k=0}^{\infty }{\gamma }^{k}{r}_{t+k},$$

where $\gamma$ is the discount factor, and ${r}_{t}$ is the reward at time point $t$. The agent learns and updates state-action values, which were initialized to 0 according to

$$Q\left({s}_{t},{a}_{t}\right)\leftarrow Q\left({s}_{t},{a}_{t}\right)+\alpha {\delta }_{t},$$

where $Q\left({s}_{t},{a}_{t}\right)$ is the state-action value for action $a$ in state $s$ at time point $t$, $\alpha$ is the learning rate, and ${\delta }_{t}$ is the TD error. The TD error for the on-policy SARSA agent is

$${{\delta }_{t}=r}_{t+1}+\gamma Q\left({s}_{t+1},{a}_{t+1}\right)-Q\left({s}_{t},{a}_{t}\right),$$

where ${r}_{t+1}$ is the reward at the next time point, $\gamma$ is the discount factor, and $Q\left({s}_{t+1},{a}_{t+1}\right)$ is the state-action value at the next time point using the next state and chosen action. The TD error for the off-policy Q-learning agent is

$${\delta }_{t}={r}_{t+1}+\gamma \mathop{\max }\limits_{a}Q\left({s}_{t+1},a\right)-Q\left({s}_{t},{a}_{t}\right),$$

where, in contrast to SARSA, the Q-learning agent uses the maximum of the state-action values in the next state $\mathop{\max }\limits_{a}Q\left({s}_{t+1},a\right)$.

Actions only influenced the probability of state transitions in the cue epoch. In the other states, a dummy action was selected, and the action values were set to the same value for each action after the update. RPEs were modeled as the TD errors of entering a state.

For action selection, we used the ‘softmax’ choice rule, which transforms the *Q* values to probabilities of choosing an action according to

$$P\left(a={{\mathrm{left}}}\right)=\frac{\exp [\,\beta (Q_{{{\mathrm{left}}}}+{\beta }_{{{\mathrm{bias}}}{{\_}}{{\mathrm{left}}}})]}{\exp [\,\beta (Q_{{{\mathrm{left}}}}+{\beta }_{{{\mathrm{bias}}}{{\_}}{{\mathrm{left}}}})]+\exp [\,\beta (Q_{{{\mathrm{right}}}}+{\beta }_{{{\mathrm{bias}}}{{\_}}{{\mathrm{right}}}})]},$$

where $\beta$ is the inverse temperature that controls the stochasticity of choice, ranging from 0 (random choice) to $\infty$ (deterministic choice of the highest value), and ${\beta }_{{{\mathrm{bias}}}}$ is a bias parameter that was arbitrarily initialized with initial value ${\beta }_{{{\mathrm{bias}}}0}$ for one of the two actions (and 0 for the other action) for each agent and set to exponentially decay to 0 across sessions with decay constant ${\lambda }_{{{\mathrm{bias}}}}$. To account for the animals’ response biases (Fig. 1f), the bias term in the model was used during task rules with initial learning (location task) or when the performance dropped below chance level due to perseveration on the previous rule (frequency reversed task). We thus assumed that the response bias originated in the action selection system, not the valuation system.

The rule (that is, the environment) was switched after two sessions with criterion performance, as in the behavioral experiments. To model the resulting effects on behavioral and dopamine signatures, we explored different variants of resetting *Q* values after the rule switch: no reset, complete reset and partial reset, where cue-state *Q* values, which represent the choice-guiding information learned during the previous task rule, were retained.

We simulated 300 trials per session. The following parameters were used for all simulations presented in the figures: $\alpha =0.003$, $\gamma =1$, $\beta =6$, ${\beta }_{{{\mathrm{bias}}}0}=0.4$ and ${\lambda }_{{{\mathrm{bias}}}}=0.4$.

State RPEs were obtained from the SARSA partial Q reset model that was generated using the same settings as described earlier. State values were updated according to

$$V\left({s}_{t}\right)\leftarrow V\left({s}_{t}\right)+\alpha {\delta }_{t},$$

where $V\left({s}_{t},{a}_{t}\right)$ is the state value in state $s$ at time point $t$, $\alpha$ is the learning rate, and ${\delta }_{t}$ is the TD error

$${{\delta }_{t}=r}_{t+1}+\gamma V\left({s}_{t+1}\right)-V\left({s}_{t}\right).$$

For state value learning, we applied the same state space and partial value reset as for state-action value learning and used the following parameters: $\alpha =0.015$ and $\gamma =1$.

### Reporting summary

Further information on research design is available in the Nature Portfolio Reporting Summary linked to this article.

## Online content

Any methods, additional references, Nature Portfolio reporting summaries, source data, extended data, supplementary information, acknowledgements, peer review information; details of author contributions and competing interests; and statements of data and code availability are available at 10.1038/s41593-023-01567-2.

## Supplementary information

Supplementary Information Supplementary Tables 1 and 2.

Reporting Summary

## Source data

Source Data Fig. 1 Statistical source data.

Source Data Fig. 2 Statistical source data.

Source Data Fig. 3 Statistical source data.

Source Data Fig. 4 Statistical source data.

Source Data Fig. 5 Statistical source data.

Source Data Fig. 6 Statistical source data.

Source Data Extended Data Fig. 1 Statistical source data.

Source Data Extended Data Fig. 2 Statistical source data.

Source Data Extended Data Fig. 3 Statistical source data.

Source Data Extended Data Fig. 4 Statistical source data.

Source Data Extended Data Fig. 5 Statistical source data.

Source Data Extended Data Fig. 6 Statistical source data.

## Data Availability

Raw data are available on request from the authors. Source data are provided with this paper.

## Code availability

Code is available on request from the authors.
