# Supplementary material for: Striatal dopamine signals reflect perceived cue–action–outcome associations in mice
Source: Nat Neurosci. 2024 Jan 30;27(4):747–57. doi: 10.1038/s41593-023-01567-2 (PMC11001585; doi:10.1038/s41593-023-01567-2)
Supplement: Supplementary file 1 — Supplementary Tables 1 and 2. [file 41593_2023_1567_MOESM1_ESM.pdf]

nature neuroscience

Article

https://doi.org/10.1038/s41593-023-01567-2

Striatal dopamine signals reflect perceived cue–action–outcome associations in mice

In the format provided by the authors and unedited

Supplementary Table 1 | Statistical tests in Fig. 2 and Fig. 3. KW, Kruskal-Wallis H-test; PCS, Pearson's chi-squared test; WRS, Wilcoxon rank-sum test; *n*, sample size (per group, if applicable); *P* (corr.), *P* value (after correction for multiple comparisons, if applicable)

Fig.	Comparison (see figure legend for details)	Test	*n*	*P* (corr.)
2d	Performance levels (VS, loc., cue)	KW	12, 15, 16	0.006
2d	Performance levels (VS, loc., outcome)	KW	12, 15, 16	2.5×10^{-7}
2d	Performance levels (VS, freq., cue)	KW	6, 9, 12	0.144
2d	Performance levels (VS, freq., outcome)	KW	6, 9, 12	1.5×10^{-4}
2d	Performance levels (VS, freq. rev., cue)	KW	6, 9, 34	0.003
2d	Performance levels (VS, freq. rev., outcome)	KW	6, 9, 34	2.5×10^{-7}
2f	Performance levels (DMS, loc., cue)	KW	10, 22, 12	0.237
2f	Performance levels (DMS, loc., outcome)	KW	10, 22, 12	0.007
2f	Performance levels (DMS, freq., cue)	KW	5, 12, 10	0.457
2f	Performance levels (DMS, freq., outcome)	KW	5, 12, 10	1.4×10^{-4}
2f	Performance levels (DMS, freq. rev., cue)	KW	10, 11, 40	0.017
2f	Performance levels (DMS, freq. rev, outcome)	KW	10, 11, 40	2.1×10^{-5}
2h	Performance levels (DLS, loc., cue)	KW	12, 24, 8	0.045
2h	Performance levels (DLS, loc., outcome)	KW	12, 24, 8	3.6×10^{-6}
2h	Performance levels (DLS, freq., cue)	KW	5, 11, 8	0.464
2h	Performance levels (DLS, freq., outcome)	KW	5, 11, 8	2.4×10^{-4}
2h	Performance levels (DLS, freq. rev., cue)	KW	11, 14, 33	0.270
2h	Performance levels (DLS, freq. rev., outcome)	KW	11, 14, 33	4.2×10^{-10}
4d	Stay vs. switch conditions (VS, L^{last}, performance)	PCS	755, 724; 89, 118	0.180
4d	Stay vs. switch conditions (VS, F^1, performance)	PCS	1038, 259; 143, 927	2.2×10^{-227}
4d	Stay vs. switch conditions (VS, F^2, performance)	PCS	1043, 580, 180, 652	7.1×10^{-88}
4d	Stay vs. switch conditions (VS, F^3, performance)	PCS	829, 611; 63, 278	1.7×10^{-37}
4d	Stay vs. switch conditions (VS, F^{last}, performance)	PCS	897, 798; 52, 148	5.1×10^{-12}
4d	Stay vs. switch conditions (VS, L^{last}, cue)	WRS	755, 724	1.0
4d	Stay vs. switch conditions (VS, F^1, cue)	WRS	1038, 259	5.3×10^{-7}
4d	Stay vs. switch conditions (VS, F^2, cue)	WRS	1043, 580	2.0×10^{-29}
4d	Stay vs. switch conditions (VS, F^3, cue)	WRS	829, 611	1.1×10^{-25}
4d	Stay vs. switch conditions (VS, F^{last}, cue)	WRS	897, 798	6.6×10^{-22}
4d	Stay vs. switch conditions (VS, L^{last}, outcome)	WRS	755, 724	1.0
4d	Stay vs. switch conditions (VS, F^1, outcome)	WRS	1038, 259	1.0
4d	Stay vs. switch conditions (VS, F^2, outcome)	WRS	1043, 580	6.1×10^{-28}
4d	Stay vs. switch conditions (VS, F^3, outcome)	WRS	829, 611	5.8×10^{-22}
4d	Stay vs. switch conditions (VS, F^{last}, outcome)	WRS	897, 798	9.0×10^{-7}
4e	Stay vs. switch conditions (DMS, L^{last}, performance)	PCS	572, 555; 76, 92	1.0
4e	Stay vs. switch conditions (DMS, F^1, performance)	PCS	829, 182; 143, 791	1.5×10^{-188}
4e	Stay vs. switch conditions (DMS, F^2, performance)	PCS	677, 345; 82, 419	1.4×10^{-73}
4e	Stay vs. switch conditions (DMS, F^3, performance)	PCS	714, 490; 78, 297	1.0×10^{-37}
4e	Stay vs. switch conditions (DMS, F^{last}, performance)	PCS	640, 567; 22, 93	3.7×10^{-11}
4e	Stay vs. switch conditions (DMS, L^{last}, cue)	WRS	572, 555	1.0
4e	Stay vs. switch conditions (DMS, F^1, cue)	WRS	829, 182	0.402
4e	Stay vs. switch conditions (DMS, F^2, cue)	WRS	677, 345	0.028
4e	Stay vs. switch conditions (DMS, F^3, cue)	WRS	714, 490	6.6×10^{-9}
4e	Stay vs. switch conditions (DMS, F^{last}, cue)	WRS	640, 567	0.001
4e	Stay vs. switch conditions (DMS, L^{last}, outcome)	WRS	572, 555	1.0
4e	Stay vs. switch conditions (DMS, F^1, outcome)	WRS	829, 182	1.0
4e	Stay vs. switch conditions (DMS, F^2, outcome)	WRS	677, 345	0.637
4e	Stay vs. switch conditions (DMS, F^3, outcome)	WRS	714, 490	0.004
4e	Stay vs. switch conditions (DMS, F^{last}, outcome)	WRS	640, 567	0.832
4f	Stay vs. switch conditions (DLS, L^{last}, performance)	PCS	488, 503; 92, 85	1.0
4f	Stay vs. switch conditions (DLS, F^1, performance)	PCS	651, 209; 146, 592	7.6×10^{-109}
4f	Stay vs. switch conditions (DLS, F^2, performance)	PCS	622, 404; 131, 350	1.3×10^{-32}
4f	Stay vs. switch conditions (DLS, F^3, performance)	PCS	593, 416; 85, 257	2.2×10^{-26}
4f	Stay vs. switch conditions (DLS, F^{last}, performance)	PCS	549, 476; 33, 112	3.6×10^{-11}
4f	Stay vs. switch conditions (DLS, L^{last}, cue)	WRS	488, 503	0.240
4f	Stay vs. switch conditions (DLS, F^1, cue)	WRS	651, 209	1.0
4f	Stay vs. switch conditions (DLS, F^2, cue)	WRS	622, 404	1.0,
4f	Stay vs. switch conditions (DLS, F^3, cue)	WRS	593, 416	1.0
4f	Stay vs. switch conditions (DLS, F^{last}, cue)	WRS	549, 476	1.0
4f	Stay vs. switch conditions (DLS, L^{last}, outcome)	WRS	488, 503	0.600
4f	Stay vs. switch conditions (DLS, F^1, outcome)	WRS	651, 209	1.0
4f	Stay vs. switch conditions (DLS, F^2, outcome)	WRS	622, 404	8.7×10^{-5}
4f	Stay vs. switch conditions (DLS, F^3, outcome)	WRS	593, 416	0.001
4f	Stay vs. switch conditions (DLS, F^{last}, outcome)	WRS	549, 476	2.2×10^{-5}

Supplementary Table 2 | Linear mixed modeling analyses. All analyses were performed with animal identity as grouping factor for random intercept and random slope for the independent variable. DV, dependent variable; IV, independent variable; Coef., coefficient for IV; *t*, test statistic, *P*, *P* value, *P* (corr.), *P* value after correction for multiple comparisons (if applicable).

Fig.	Description	DV	IV	Coef.	*t*	*P*	*P* (corr.)
2d	VS, loc.	Cue DA	Session performance	-1.038	-1.973	4.85×10^{-2}	4.85×10^{-2}
2d	VS, loc.	Out. DA	Session performance	-7.515	-17.066	2.67×10^{-65}	2.67×10^{-65}
2d	VS, freq.	Cue DA	Session performance	-1.120	-1.900	5.75×10^{-2}	5.75×10^{-2}
2d	VS, freq.	Out. DA	Session performance	-7.187	-14.270	3.38×10^{-46}	3.38×10^{-46}
2d	VS, freq. rev.	Cue DA	Session performance	-2.901	-3.581	3.43×10^{-4}	3.43×10^{-4}
2d	VS, freq. rev.	Out. DA	Session performance	-7.789	-7.795	6.44×10^{-15}	6.44×10^{-15}
2f	DMS, loc.	Cue DA	Session performance	0.574	0.677	4.98×10^{-1}	4.98×10^{-1}
2f	DMS, loc.	Out. DA	Session performance	-2.272	-4.034	5.47×10^{-5}	5.47×10^{-5}
2f	DMS, freq.,	Cue DA	Session performance	-0.415	-0.750	4.53×10^{-1}	4.53×10^{-1}
2f	DMS, freq.	Out. DA	Session performance	-1.872	-5.370	7.88×10^{-8}	7.88×10^{-8}
2f	DMS, freq. rev.	Cue DA	Session performance	-1.206	-2.577	9.97×10^{-3}	9.97×10^{-3}
2f	DMS, freq. rev.	Out. DA	Session performance	-1.437	-8.095	5.74×10^{-16}	5.74×10^{-16}
2h	DLS, loc.	Cue DA	Session performance	0.897	2.812	4.92×10^{-3}	4.92×10^{-3}
2h	DLS, loc.	Out. DA	Session performance	-7.965	-5.945	2.76×10^{-9}	2.76×10^{-9}
2h	DLS, freq.	Cue DA	Session performance	0.046	0.183	8.55×10^{-1}	8.55×10^{-1}
2h	DLS, freq.	Out. DA	Session performance	-8.589	-7.024	2.15×10^{-12}	2.15×10^{-12}
2h	DLS, freq. rev.	Cue DA	Session performance	0.257	1.358	1.75×10^{-1}	1.75×10^{-1}
2h	DLS, freq. rev.	Out. DA	Session performance	-7.333	-16.449	8.46×10^{-61}	8.46×10^{-61}
3a	VS	Performance	Novice early/late	-0.025	-3.097	1.96×10^{-3}	1.96×10^{-3}
3a	VS	Cue DA	Novice early/late	-0.868	-3.593	3.27×10^{-4}	3.27×10^{-4}
3a	VS	Spout DA	Novice early/late	4.011	5.731	9.96×10^{-9}	9.96×10^{-9}
3a	VS	Out. DA	Novice early/late	-0.106	-0.343	7.32×10^{-1}	7.32×10^{-1}
3b	DMS	Performance	Novice early/late	-0.021	-1.122	2.62×10^{-1}	2.62×10^{-1}
3b	DMS	Cue DA	Novice early/late	-0.945	-2.307	2.11×10^{-2}	2.11×10^{-2}
3b	DMS	Spout DA	Novice early/late	1.083	5.167	2.38×10^{-7}	2.38×10^{-7}
3b	DMS	Out. DA	Novice early/late	0.082	0.240	8.11×10^{-1}	8.11×10^{-1}
3c	DLS	Performance	Novice early/late	-0.029	-2.080	3.75×10^{-2}	3.75×10^{-2}
3c	DLS	Cue DA	Novice early/late	0.052	0.505	6.14×10^{-1}	6.14×10^{-1}
3c	DLS	Spout DA	Novice early/late	1.551	3.905	9.42×10^{-5}	9.42×10^{-5}
3c	DLS	Out. DA	Novice early/late	-1.150	-1.907	5.65×10^{-2}	5.65×10^{-2}
3d	VS, nov.	Cue DA	Correct/error	1.772	2.535	1.12×10^{-2}	3.37×10^{-2}
3d	VS, int.	Cue DA	Correct/error	0.218	1.179	2.38×10^{-1}	7.15×10^{-1}
3d	VS, exp.	Cue DA	Correct/error	0.268	1.010	3.13×10^{-1}	9.38×10^{-1}
3e	DMS, nov.	Cue DA	Correct/error	0.912	3.040	2.37×10^{-3}	7.10×10^{-3}
3e	DMS, int.	Cue DA	Correct/error	0.380	1.124	2.61×10^{-1}	7.83×10^{-1}
3e	DMS, exp.	Cue DA	Correct/error	0.195	1.052	2.93×10^{-1}	8.78×10^{-1}
3f	DLS, nov.	Cue DA	Correct/error	0.296	1.849	6.45×10^{-2}	1.93×10^{-1}
3f	DLS, int.	Cue DA	Correct/error	0.328	2.786	5.34×10^{-3}	1.60×10^{-2}
3f	DLS, exp.	Cue DA	Correct/error	0.127	0.552	5.81×10^{-1}	1.00×10^{0}
4d	VS, L^{last}	Performance	Stay/switch	0.035	1.697	1.33×10^{-1}	6.66×10^{-1}
4d	VS, F^{1}	Performance	Stay/switch	0.652	12.910	4.94×10^{-5}	2.47×10^{-4}
4d	VS, F^{2}	Performance	Stay/switch	0.383	8.956	2.61×10^{-4}	1.31×10^{-3}
4d	VS, F^{3}	Performance	Stay/switch	0.247	7.269	6.02×10^{-4}	3.01×10^{-3}
4d	VS, F^{last}	Performance	Stay/switch	0.098	5.330	7.85×10^{-4}	3.92×10^{-3}
4d	VS, L^{last}	Cue DA	Stay/switch	-0.104	-0.603	5.46×10^{-1}	1.00×10^{0}
4d	VS, F^{1}	Cue DA	Stay/switch	0.671	4.916	8.85×10^{-7}	4.42×10^{-6}
4d	VS, F^{2}	Cue DA	Stay/switch	1.078	5.864	4.52×10^{-9}	2.26×10^{-8}
4d	VS, F^{3}	Cue DA	Stay/switch	1.088	6.069	1.29×10^{-9}	6.43×10^{-9}
4d	VS, F^{last}	Cue DA	Stay/switch	0.839	6.103	1.04×10^{-9}	5.21×10^{-9}
4d	VS, L^{last}	Out. DA	Stay/switch	-0.065	-0.561	5.74×10^{-1}	1.00×10^{0}
4d	VS, F^{1}	Out. DA	Stay/switch	-0.245	-1.396	1.63×10^{-1}	8.13×10^{-1}
4d	VS, F^{2}	Out. DA	Stay/switch	-1.116	-2.972	2.96×10^{-3}	1.48×10^{-2}
4d	VS, F^{3}	Out. DA	Stay/switch	-0.974	-5.802	6.56×10^{-9}	3.28×10^{-8}
4d	VS, F^{last}	Out. DA	Stay/switch	-0.493	-5.638	1.72×10^{-8}	8.59×10^{-8}
4e	DMS, L^{last}	Performance	Stay/switch	0.027	0.642	5.56×10^{-1}	1.00×10^{0}
4e	DMS, F^{1}	Performance	Stay/switch	0.658	10.697	4.30×10^{-4}	2.15×10^{-3}
4e	DMS, F^{2}	Performance	Stay/switch	0.438	10.571	4.10×10^{-4}	2.05×10^{-3}
4e	DMS, F^{3}	Performance	Stay/switch	0.281	3.403	2.68×10^{-2}	1.34×10^{-1}
4e	DMS, F^{last}	Performance	Stay/switch	0.109	3.999	1.42×10^{-2}	7.10×10^{-2}
4e	DMS, L^{last}	Cue DA	Stay/switch	0.087	0.429	6.68×10^{-1}	1.00×10^{0}
4e	DMS, F^{1}	Cue DA	Stay/switch	0.370	1.835	6.64×10^{-2}	3.32×10^{-1}
4e	DMS, F^{2}	Cue DA	Stay/switch	0.557	1.225	2.20×10^{-1}	1.00×10^{0}
4e	DMS, F^{3}	Cue DA	Stay/switch	1.001	2.429	1.51×10^{-2}	7.57×10^{-2}
4e	DMS, F^{last}	Cue DA	Stay/switch	0.571	1.665	9.59×10^{-2}	4.79×10^{-1}
4e	DMS, L^{last}	Out. DA	Stay/switch	-0.031	-0.350	7.26×10^{-1}	1.00×10^{0}
4e	DMS, F^{1}	Out. DA	Stay/switch	0.081	0.569	5.69×10^{-1}	1.00×10^{0}
4e	DMS, F^{2}	Out. DA	Stay/switch	-0.121	-1.303	1.92×10^{-1}	9.62×10^{-1}
4e	DMS, F^{3}	Out. DA	Stay/switch	-0.334	-1.456	1.46×10^{-1}	7.28×10^{-1}
4e	DMS, F^{last}	Out. DA	Stay/switch	-0.155	-0.860	3.90×10^{-1}	1.00×10^{0}
4f	DLS, L^{last}	Performance	Stay/switch	-0.020	-0.318	7.72×10^{-1}	1.00×10^{0}

4f	DLS, F^1	Performance	Stay/switch	0.548	7.213	5.47×10^{-3}	2.73×10^{-2}
4f	DLS, F^2	Performance	Stay/switch	0.289	4.023	2.75×10^{-2}	1.38×10^{-1}
4f	DLS, F^3	Performance	Stay/switch	0.256	4.639	1.71×10^{-2}	8.57×10^{-2}
4f	DLS, F^{last}	Performance	Stay/switch	0.129	4.399	1.63×10^{-2}	8.13×10^{-2}
4f	DLS, L^{last}	Cue DA	Stay/switch	-0.201	-1.443	1.49×10^{-1}	7.44×10^{-1}
4f	DLS, F^1	Cue DA	Stay/switch	-0.120	-0.612	5.40×10^{-1}	1.00×10^{0}
4f	DLS, F^2	Cue DA	Stay/switch	-0.152	-0.912	3.62×10^{-1}	1.00×10^{0}
4f	DLS, F^3	Cue DA	Stay/switch	0.088	0.719	4.72×10^{-1}	1.00×10^{0}
4f	DLS, F^{last}	Cue DA	Stay/switch	-0.115	-0.656	5.12×10^{-1}	1.00×10^{0}
4f	DLS, L^{last}	Out. DA	Stay/switch	-0.205	-0.976	3.29×10^{-1}	1.00×10^{0}
4f	DLS, F^1	Out. DA	Stay/switch	-0.185	-0.555	5.79×10^{-1}	1.00×10^{0}
4f	DLS, F^2	Out. DA	Stay/switch	-0.692	-2.345	1.90×10^{-2}	9.51×10^{-2}
4f	DLS, F^3	Out. DA	Stay/switch	-0.615	-3.205	1.35×10^{-3}	6.76×10^{-3}
4f	DLS, F^{last}	Out. DA	Stay/switch	-0.659	-2.786	5.34×10^{-3}	2.67×10^{-2}
5a	VS, loc., corr.	Out. DA	Session performance	-7.198	-13.310	2.04×10^{-40}	2.04×10^{-40}
5a	VS, freq., corr.	Out. DA	Session performance	-7.248	-13.963	2.61×10^{-44}	2.61×10^{-44}
5a	VS, freq. rev., corr.	Out. DA	Session performance	-7.317	-5.536	3.10×10^{-8}	3.10×10^{-8}
5b	DMS, loc., corr.	Out. DA	Session performance	-2.256	-3.687	2.27×10^{-4}	2.27×10^{-4}
5b	DMS freq., corr.	Out. DA	Session performance	-1.872	-5.370	7.88×10^{-8}	7.88×10^{-8}
5b	DMS, freq. rev., corr.	Out. DA	Session performance	-1.276	-3.403	6.67×10^{-4}	6.67×10^{-4}
5c	DLS, loc., corr.	Out. DA	Session performance	-5.253	-6.787	1.15×10^{-11}	1.15×10^{-11}
5c	DLS freq., corr.	Out. DA	Session performance	-8.620	-7.809	5.77×10^{-15}	5.77×10^{-15}
5c	DLS, freq. rev., corr.	Out. DA	Session performance	-6.528	-14.232	5.79×10^{-46}	5.79×10^{-46}
5a	VS, loc., error	Out. DA	Session performance	0.147	0.532	5.95×10^{-1}	5.95×10^{-1}
5a	VS, freq., error	Out. DA	Session performance	1.397	2.363	1.81×10^{-2}	1.81×10^{-2}
5a	VS, freq. rev., error	Out. DA	Session performance	-0.337	-1.130	2.59×10^{-1}	2.59×10^{-1}
5b	DMS, loc., error	Out. DA	Session performance	1.622	2.037	4.16×10^{-2}	4.16×10^{-2}
5b	DMS, freq., error	Out. DA	Session performance	1.213	1.277	2.02×10^{-1}	2.02×10^{-1}
5b	DMS, freq. rev., error	Out. DA	Session performance	1.362	2.253	2.43×10^{-2}	2.43×10^{-2}
5c	DLS, loc., error	Out. DA	Session performance	0.734	1.717	8.60×10^{-2}	8.60×10^{-2}
5c	DLS, freq., error	Out. DA	Session performance	0.268	0.369	7.12×10^{-1}	7.12×10^{-1}
5c	DLS, freq. rev., error	Out. DA	Session performance	-0.131	-0.318	7.51×10^{-1}	7.51×10^{-1}
5e	VS, corr.	Out. DA	Session performance	-1.278	-2.138	3.25×10^{-2}	3.25×10^{-2}
5e	VS, error	Out. DA	Session performance	-0.277	-0.867	3.86×10^{-1}	3.86×10^{-1}
5e	VS, prob., low	Out. DA	Error/corr. but no rew.	-0.580	-2.746	6.03×10^{-3}	1.81×10^{-2}
5e	VS, prob., high	Out. DA	Error/corr. but no rew.	0.195	0.917	3.59×10^{-1}	1.00×10^{0}
5e	VS, prob, low	Out. DA	Error/corr. but no rew.	-0.499	-1.616	1.06×10^{-1}	3.18×10^{-1}
