# Supplementary material for: Striatal dopamine signals reflect perceived cue–action–outcome associations in mice
Source: Nat Neurosci. 2024 Jan 30;27(4):747–57. doi: 10.1038/s41593-023-01567-2 (PMC11001585; doi:10.1038/s41593-023-01567-2)
Supplement: Supplementary file 2 — Reporting Summary [file 41593_2023_1567_MOESM2_ESM.pdf]

nature portfolio

Corresponding author(s): Simon Jacob

Last updated by author(s): Dec 5, 2023

Reporting Summary

Nature Portfolio wishes to improve the reproducibility of the work that we publish. This form provides structure for consistency and transparency in reporting. For further information on Nature Portfolio policies, see our Editorial Policies and the Editorial Policy Checklist.

Statistics

For all statistical analyses, confirm that the following items are present in the figure legend, table legend, main text, or Methods section.

n/a	Confirmed	
☐	☒	The exact sample size (n) for each experimental group/condition, given as a discrete number and unit of measurement
☐	☒	A statement on whether measurements were taken from distinct samples or whether the same sample was measured repeatedly
☐	☒	The statistical test(s) used AND whether they are one- or two-sided<br>*Only common tests should be described solely by name; describe more complex techniques in the Methods section.*
☐	☒	A description of all covariates tested
☐	☒	A description of any assumptions or corrections, such as tests of normality and adjustment for multiple comparisons
☐	☒	A full description of the statistical parameters including central tendency (e.g. means) or other basic estimates (e.g. regression coefficient) AND variation (e.g. standard deviation) or associated estimates of uncertainty (e.g. confidence intervals)
☐	☒	For null hypothesis testing, the test statistic (e.g. F, t, r) with confidence intervals, effect sizes, degrees of freedom and P value noted<br>*Give P values as exact values whenever suitable.*
☒	☐	For Bayesian analysis, information on the choice of priors and Markov chain Monte Carlo settings
☐	☒	For hierarchical and complex designs, identification of the appropriate level for tests and full reporting of outcomes
☒	☐	Estimates of effect sizes (e.g. Cohen's d, Pearson's r), indicating how they were calculated

Our web collection on statistics for biologists contains articles on many of the points above.

Software and code

Policy information about availability of computer code

Data collection	For surgeries we used automated navigation software Neurostar StereoDrive (version 3.1.5). For behavioral and photometric data collection we used Matlab (version R2018a), the Matlab toolbox MonkeyLogic (version 2, build 206), and Plexon OmniPlex (version 1.18.3).
Data analysis	For data analysis we used Python (version 3.6.8) and the Python packages NumPy (version 1.16.4), SciPy (version 1.5.4), pandas (version 0.25.1), scikit-learn (version 0.22.1), statsmodels (version 0.11.1), pymer4 (version 0.8.0.9001), psytrack, and pyglmnet (version 1.1).

For manuscripts utilizing custom algorithms or software that are central to the research but not yet described in published literature, software must be made available to editors and reviewers. We strongly encourage code deposition in a community repository (e.g. GitHub). See the Nature Portfolio guidelines for submitting code & software for further information.

Data

Policy information about availability of data

All manuscripts must include a data availability statement. This statement should provide the following information, where applicable:

- Accession codes, unique identifiers, or web links for publicly available datasets
- A description of any restrictions on data availability
- For clinical datasets or third party data, please ensure that the statement adheres to our policy

Raw data are available on request from the authors. Source Data are provided with this paper.

Human research participants

Policy information about studies involving human research participants and Sex and Gender in Research.

Reporting on sex and gender	Not applicable
Population characteristics	Not applicable
Recruitment	Not applicable
Ethics oversight	Not applicable

Note that full information on the approval of the study protocol must also be provided in the manuscript.

Field-specific reporting

Please select the one below that is the best fit for your research. If you are not sure, read the appropriate sections before making your selection.

☒ Life sciences ☐ Behavioural & social sciences ☐ Ecological, evolutionary & environmental sciences

For a reference copy of the document with all sections, see nature.com/documents/nr-reporting-summary-flat.pdf

Life sciences study design

All studies must disclose on these points even when the disclosure is negative.

Sample size	No statistical methods were used to pre-determine sample sizes, but our sample sizes are similar to those reported in previous publications (see Methods). Datasets were completed before data analysis and no data were added at later timepoints.
Data exclusions	As reported in Methods, one animal was excluded from dLight analysis due to low signal amplitude and two animals were lost during training and completed only the first two tasks.
Replication	No additional replication measures were performed, except succesful replication of main results across individual animals (4 to 6 animals).
Randomization	All animals underwent the same behavioral protocol. There was no random assignment to experimental groups.
Blinding	We did not perform blinded experiments, since all animals underwent the same behavioral protocol.

Reporting for specific materials, systems and methods

We require information from authors about some types of materials, experimental systems and methods used in many studies. Here, indicate whether each material, system or method listed is relevant to your study. If you are not sure if a list item applies to your research, read the appropriate section before selecting a response.

Materials & experimental systems

n/a	Involved in the study
☒	☐ Antibodies
☒	☐ Eukaryotic cell lines
☒	☐ Palaeontology and archaeology
☐	☒ Animals and other organisms
☒	☐ Clinical data
☒	☐ Dual use research of concern

Methods

n/a	Involved in the study
☒	☐ ChIP-seq
☒	☐ Flow cytometry
☒	☐ MRI-based neuroimaging

Animals and other research organisms

Policy information about studies involving animals; ARRIVE guidelines recommended for reporting animal research, and Sex and Gender in Research

Laboratory animals	Wild-type male mice (C57BL/6J, Charles River) were used. Mice were 8-10 weeks old at the beginning of the experiments.

Wild animals	No wild animals were used.
Reporting on sex	Only male mice were used. There is no indication that the effects are sex-specific.
Field-collected samples	No field-collected samples were used.
Ethics oversight	All animal procedures were authorized by the local government (Regierung von Oberbayern, license number ROB-55.2-2532.Vet_02-17-119).

Note that full information on the approval of the study protocol must also be provided in the manuscript.
